# Genomic Characterization of *Burkholderia pseudomallei* Isolates Selected for Medical Countermeasures Testing: Comparative Genomics Associated with Differential Virulence

**DOI:** 10.1371/journal.pone.0121052

**Published:** 2015-03-24

**Authors:** Jason W. Sahl, Christopher J. Allender, Rebecca E. Colman, Katy J. Califf, James M. Schupp, Bart J. Currie, Kristopher E. Van Zandt, H. Carl Gelhaus, Paul Keim, Apichai Tuanyok

**Affiliations:** 1 Department of Pathogen Genomics, Translational Genomics Research Institute, Flagstaff, Arizona, United States of America; 2 Center for Microbial Genetics and Genomics, Northern Arizona University, Flagstaff, Arizona, United States of America; 3 Department of Tropical and Emerging Infectious Diseases, Menzies School of Health Research, Casuarina NT, Australia; 4 Battelle Biomedical Research Center (BBRC), Columbus, Ohio, United States of America; 5 Department of Tropical Medicine, Medical Microbiology and Pharmacology, and Pacific Center for Emerging Infections Diseases Research, University of Hawaii at Manoa, Honolulu, Hawaii, United States of America; University of Toledo School of Medicine, UNITED STATES

## Abstract

*Burkholderia pseudomallei* is the causative agent of melioidosis and a potential bioterrorism agent. In the development of medical countermeasures against *B*. *pseudomallei* infection, the US Food and Drug Administration (FDA) animal Rule recommends using well-characterized strains in animal challenge studies. In this study, whole genome sequence data were generated for 6 *B*. *pseudomallei* isolates previously identified as candidates for animal challenge studies; an additional 5 isolates were sequenced that were associated with human inhalational melioidosis. A core genome single nucleotide polymorphism (SNP) phylogeny inferred from a concatenated SNP alignment from the 11 isolates sequenced in this study and a diverse global collection of isolates demonstrated the diversity of the proposed Animal Rule isolates. To understand the genomic composition of each isolate, a large-scale blast score ratio (LS-BSR) analysis was performed on the entire pan-genome; this demonstrated the variable composition of genes across the panel and also helped to identify genes unique to individual isolates. In addition, a set of ~550 genes associated with pathogenesis in *B*. *pseudomallei* were screened against the 11 sequenced genomes with LS-BSR. Differential gene distribution for 54 virulence-associated genes was observed between genomes and three of these genes were correlated with differential virulence observed in animal challenge studies using BALB/c mice. Differentially conserved genes and SNPs associated with disease severity were identified and could be the basis for future studies investigating the pathogenesis of *B*. *pseudomallei*. Overall, the genetic characterization of the 11 proposed Animal Rule isolates provides context for future studies involving *B*. *pseudomallei* pathogenesis, differential virulence, and efficacy to therapeutics.

## Introduction

*Burkholderia pseudomallei* is a pathogen endemic to Southeast Asia and Northern Australia but is increasingly found in other parts of the world including India, South America, and Africa, where it is naturally found in soil and water [1]. The bacterium is the causative agent of melioidosis [2–5], a potentially fatal disease in humans. *B*. *pseudomallei* is also considered to be a Tier 1 biothreat agent due to its ease of attainment, ability to cause lethal disease, intrinsic antibiotic resistance [6], and lack of a melioidosis vaccine [7]. The development of appropriate medical countermeasures against melioidosis has been hampered by access to human patients for clinical trials with compounds that are not currently approved for the treatment of melioidosis. To address this concern, the US Food and Drug Administration (FDA) has instituted the “Animal Rule” 21 CFR that calls for well-characterized strains to be used in animal challenge studies [8], including BALB/c mice, which have shown to represent acute human melioidosis [9]. Based on several selection criteria, a recent study selected a panel of six *B*. *pseudomallei* strains that would be appropriate for challenge studies under the FDA Animal Rule [7].

In the current study, we used whole-genome sequencing (WGS) to genetically characterize a panel of *B*. *pseudomallei* strains to be used as challenge material in therapeutic efficacy studies under the Animal Rule. In addition, we sequenced 5 *B*. *pseudomallei* strains associated with inhalational disease for evaluation as potential challenge strains. The purpose of WGS on these isolates was to (1) characterize the genomic background in each isolate; (2) identify the phylogenetic diversity of panel isolates in the context of a global set of genomes and; (3) identify the distribution of characterized virulence factors for correlation with virulence data obtained in animal challenge studies.

## Methods

### Strain selection

Eleven diverse isolates were selected for sequencing (Table 1). Six of these isolates were previously selected as part of a proposed *B*. *pseudomallei* strain panel, based on several selection criteria [7]. For five of these isolates, there are finished genome assemblies available in public databases [10]; these genomes were sequenced to identify any mutations compared to the published genomes. The genome for an additional isolate, NCTC 13392, has previously been published [11]. An additional 5 isolates were selected based on recent isolation and suspected inhalational disease and were associated with acute pneumonia sepsis.

**Table 1:** Details of isolates sequenced in current study.

Isolate	Isolation source	Isolation country	Isolation Year	Passages	SRA	Genbank Accession	BEI accession
1106a*	liver abscess	Thailand	1993	10	SRX263957	N/A	NR-44208
K96243*	blood	Thailand	1996	22	SRR797065	N/A	NR-44206
MSHR305*	brain	Australia	1994	10	SRX263963	N/A	NR-44225
MSHR668*	blood	Australia	1995	11	SRX259746	N/A	NR-44224
406e*	toe swab	Thailand	1988	10	SRX256398	AQTK00000000	NR-44207
NCTC 13392*	unknown	Thailand	1996	unknown	SRX245558	AOUG00000000	N/A
1026b*	blood	Thailand	1993	9	SRX259661	N/A	NR-9910
MSHR5855	sputum	Australia	2011	7	SRX526461	JMMV00000000	NR-45120
MSHR5858	sputum	Australia	2011	2	SRX264496	AVAK00000000	NR-45120
HBPUB10134a	sputum	Thailand	2010	10	SRR796658	AVAL00000000	NR-44220
HBPUB10303a	sputum	Thailand	2011	10	SRX254948	AVAM00000000	NR-44221

*genome has been sequenced previously

### Animal challenge studies

285 BALB/c mice (100% female) were purchased from Charles River Laboratories and were randomly selected and placed into challenge groups (n = 7) based on different isolates and dosing. Mice here housed in Innovive IVC mouse racks using disposable caging (7 mice per cage). Sedated mice were challenged by intranasal inoculation (15 μl per nare) of target doses diluted in Dulbecco’s Phosphate-Buffered Saline (PBS); mice were anesthetized intraperitoneally with ketamine (50–120 mg/kg) and xylazine (5–10 mg/kg). Prior to challenge, cultures were grown for 22 hours shaking at 37°C at 250xRPM; no mice were mock-treated in this study. The culture was then centrifuged and re-suspended in PBS containing 0.01% gelatin. The concentration of each challenge dilution was determined by spread plate enumeration.

Following challenge, mice were monitored every 8 hours between days 1 and 7, then twice daily between days 8 and 21; sample HBPUB10303a was only challenged for 14 days due to unforeseen delays in starting the experiment. Observations were made for clinical signs of illness, including respiratory distress, loss of appetite and activity, and seizures; any animal judged to be moribund by a trained animal technician was humanely euthanized. All study survivors were humanely euthanized with CO_2_ inhalation on Study Day 21. Kaplan-Meier survival curves were created using the ‘survival’ package in R [12]. Animal challenge studies were conducted at the Battelle Biomedical Research Center (BBRC). All animal work was approved by Battelle’s IACUC prior to study initiation.

### DNA extraction, library creation, sequencing

DNA library constructions were performed using the KAPA Library Preparation Kits with Standard PCR Library Amplification/Illumina series (KAPA biosystems, Boston MA, code KK8201). Quality and quantity of genomic DNA were evaluated by agarose gel analysis. One to two micrograms of DNA per sample were fragmented using a SonicMan (Matrical) with following parameters: 75.0 seconds pre chill, 16 cycles, 10.0 sec sonication, 100% power, 75.0 sec lid chill, 10.0 sec plate chill, and 75.0 sec post chill. The fragmented DNA was purified using QIAGEN QIAquick PCR purification columns (QIAGEN, cat. no. 28104) and eluted into 42.5 μl of Elution Buffer. The adapter ligation used 1.5 μl of the 40 μM adapter oligo mix [13]. Only one post-ligation bead cleanup was done. All purification steps were done with the 1.8x SPRI bead protocol in the KAPA protocol. Size selection of fragments was gel based; 30 μl of clean ligated material was run onto a 2% agarose gel. Several gel slices, corresponding to different average DNA fragment sizes (300, 600, and 1000bp fragments) were extracted from the gel and purified with a QIAGEN Gel Extraction kit (QIAGEN, cat. no. 28704) and eluted in 30 μl of Elution Buffer. Due to the high GC content of the samples, the PCR was optimized to improve yield and genomic coverage. Two microliters of DNA, 2 μl of 10 μM of both primers, 25 μl of NEBNext High-Fidelity 2X PCR Master Mix (New England Biolabs, Ipswich, MA, cat. no. M0541S), and 22 μl of 5 M Betaine (Sigma-Aldrich, St. Louis, MO, cat. no. B0300-1VL) were combined. The following PCR parameters were used: initial denaturation of 2 min at 98°C, 12 cycles of 30 sec at 98°C, 20 sec at 65°C, 30 sec at 72°C, with a final extension of 5 min at 72°C.

### Genome assembly

For strains that have been sequenced previously, a comparative assembly approach was employed. Reads were assembled against the reference genome (S1 Table) with AMOScmp [14]. Assembled contigs were then aligned against the reference genome with ABACAS [15] to obtain a genomic scaffold. Gaps in scaffolds were filled with IMAGE [16], which also splits un-filled scaffolds into contigs. In addition to the comparative assembly, reads were also assembled with Abyss v. 1.3.4 [17]. The two assemblies were aligned with Mugsy [18] and regions specific to the *de novo* assembly were parsed from the MAF file [19], as has been done previously [20]. Putative unique regions in the *de novo* assembly were aligned against the comparative assembly with BLASTN [21]. Regions that significantly aligned (>90% ID, >90% query length) to the comparative assembly were filtered from the analysis. Remaining regions were combined with the comparative assembly. Assembly errors were corrected from this concatenated assembly with iCORN [22], using ten iterations. For strains that had not been sequenced previously, genomes were assembled *de novo* with Abyss v 1.3.4 and assembly errors were corrected with iCORN. Assembly details are shown in S1 Table.

### *In silico* multi-locus sequence typing (*is*MLST)

BLASTN [21] was used to extract sequences from the seven loci in the *B*. *pseudomallei* MLST scheme [23] from all genome assemblies. To be considered a match, the alignment from the query genome must match a reference allele 100%. Sequence types were assigned to genomes when exact profile matches were identified. The *is*MLST functionality was performed with a custom Python script (https://gist.github.com/jasonsahl/33b0d9a8e3ac035bb92c). MLST typing information is shown in S1 Table.

### Single nucleotide polymorphism (SNP) and indel identification and annotation

For re-sequencing efforts (Table 1), raw reads were mapped to the finished genome with BWA-MEM v0.7.5 [24]. SNPs and indels were then called with the UnifiedGenotyper in GATK v. 2.7 [25]; nucmer [26] was used to find duplicate regions in the reference genome and any SNPs falling within duplicate regions were filtered from the analysis. For a SNP or indel to be called, we required a minimum coverage of 6x and a minimum proportion threshold of 0.90. Nucleotide variants were annotated with snpEFF [27]. All variants were visually confirmed from BAM files with Tablet [28].

### Synteny between previously sequenced genomes

In addition to identifying variants between finished genomes and re-sequencing projects, genome assemblies were aligned to completed genomes with MUMmer [29] and dot plots were visualized with mummerplot to identify any structural variation.

### Core genome SNP phylogeny

To visualize the phylogenetic diversity of genomes sequenced in this study, a core genome phylogenetic approach was employed; core regions are defined as sequence conserved in all examined genomes. A diverse set of finished and draft genomes was compiled (S2 Table). Raw reads were mapped to *B*. *pseudomallei* K96243 [30] with BWA-MEM [24]. SNPs were called from each BAM file with GATK, using the EMIT_ALL_CONFIDENT_SITES method, with a minimum coverage of 6x and a minimum proportion of 0.90. For genomic assemblies, SNPs were identified from nucmer alignments. Positions in K96243 were directly mapped to the corresponding position in each query genome assembly. A matrix was generated (S1 Dataset) with NASP (http://tgennorth.github.io/NASP/) from all reference positions called and polymorphic sites were identified. SNPs that could not be called by GATK, or failed to pass the depth or proportion filters, were filtered from the matrix, as well as SNPs that fell within identified duplications. The remaining dataset consisted of 62,663 SNPs, 50,290 of them being informative. A maximum likelihood phylogeny was inferred on this dataset with RAxML v8.0.17 [31, 32] using the ASC_GTRGAMMA model and 100 bootstrap replicates. The retention index (RI) value [33] was calculated with Phangorn [34].

### SNP and homoplasy density

To identify the conservation of the reference chromosomes, as well as to potentially identify any lateral gene transfer events that may confound the phylogeny, a SNP density (SD) and homoplasy density (HD) approach was employed. The SNP matrix was parsed over 1-kb non-overlapping windows of each chromosome and the number of informative SNPs was then calculated. The dataset was then processed with Paup v4.0b10 [35] to calculate the retention index (RI) value for each SNP. An RI value < 0.5 was considered to be homoplasious and the number of homoplasious SNPs over the same 1-kb window was then calculated. The HD value for each 1-kb window was calculated by dividing the number of homoplasious SNPs by the total number of informative SNPs. The distribution of SD and HD across the two chromosomes in K96243 was visualized with Circos [36].

### *In silico* gene screen

A set of previously described virulence factors [1, 30, 37–42] characterized in *B*. *pseudomallei* were compiled (S3 Table). Genes were screened against the genomes sequenced in this study with a large-scale blast score ratio (LS-BSR) approach [43]. Genes were translated with BioPython (www.biopython.org) and aligned against its nucleotide sequence with TBLASTN in order to obtain the maximum alignment (reference) bit score. Each gene was then aligned against each genome with TBLASTN in order to obtain the query alignment bit score. The BSR [44] was obtained by dividing the reference bit score by the query bit score. Genes with a BSR value > 0.90 or < 0.80 in all genomes were removed from the analysis; the complete LS-BSR matrix is available as S2 Dataset. The genes were then correlated with the tree to identify phylogenetic patterns of gene presence/absence.

### Genotype and phenotype correlations

Two approaches were performed to determine if there were correlations between genomic information and survival information obtained from animal challenge studies. The survival data were split into three categories: low virulence (100% mouse survival after 21 days), intermediate virulence (<100%, >0% survival after 21 days), and high virulence (0% mouse survival after 21 days). LS-BSR values across all genomes were multiplied by 100 in order to convert all float values to integers. The adjusted LS-BSR values were then correlated with the categorical virulence data using a Kruskal-Wallis test [45] implemented in QIIME v. 1.8.0 [46]. Core genome SNP data were also correlated to categorical data with a chi-square test implemented in SciPy. P-values were corrected with the Benjamini-Hochberg correction [47]. To test for false positives, genomes were randomly assigned to two groups of equal size and the average number of SNPs unique to each group was calculated over 10 iterations.

### Unique genomic regions

In addition to screening characterized virulence genes in assembled genomes, a *de novo* approach was also performed. All coding regions (CDSs) from all genomes in the phylogeny were compared with LS-BSR. Regions were determined to be unique to a given genome if they contained a BSR < 0.4 in all non-targeted genomes. Each unique CDS was then aligned against the GenBank [48] nucleotide database with BLASTN, and the closest hit, based on highest bit score, was identified.

### Ethics Statement

The animal protocol (2934–100007643) was approved by the Battelle Institutional Animal Care and Use Committee. The research was conducted in compliance with the Animal Welfare Act and followed the principles in the Guide for the Care and Use of Laboratory Animals from the National Research Council, Office of Laboratory Animal Welfare (OLAW), and USDA. Additionally, the research was conducted following an Institutional Animal Care and Use Committee (IACUC) approved protocol. The institution where the research was conducted is fully accredited by the Association for the Assessment and Accreditation of Laboratory Animal Care International (AAALAC).

## Results

### Comparisons of re-sequenced isolates with finished genomes

Five of the genomes sequenced in this study represent re-sequencing projects of finished genomes available in public databases (S1 Table). However, due to standard laboratory passages, new nucleotide variants can accumulate [49], and were identified in the current study using raw read data. The results demonstrate that many re-sequenced isolates show little mutation since the genomes were published (Table 2). However, the version of K96243 that was sequenced in the current study showed numerous variant positions (33) compared to the completed genome (Table 2), including the loss of two annotated stop codons. Some of these differences could be errors in the original genome sequence, which we are unable to verify. In addition to the analysis of nucleotide variants, the synteny of genomes was visualized as dot plots (S1 Fig) and demonstrated high synteny between all re-sequenced genome assemblies and finished genomes.

**Table 2:** Nucleotide variant information for re-sequencing projects conducted in current study.

Name	Chromosome	Coordinate	Reference	Query	Locus	Effect	Annotation	Proportion	Depth
K96243	NC006350.1	549058	G	A	BPSL0500	non-synonymous	hexosaminidase	0.99	165
K96243	NC006350.1	549059	T	G	BPSL0500	synonymous	hexosaminidase	1.00	163
K96243	NC006350.1	549061	C	T	BPSL0500	non-synonymous	hexosaminidase	1.00	162
K96243	NC006350.1	549062	C	T	BPSL0500	synonymous	hexosaminidase	1.00	165
K96243	NC006350.1	2399742	C	G	BPSL2010	non-synonymous	lipid metabolism-like protein	0.99	138
K96243	NC006350.1	2399743	C	G	BPSL2010	non-synonymous	lipid metabolism-like protein	0.99	140
K96243	NC006351.1	1607761	T	C	BPSS1194	non-synonymous	peptide synthase/polyketide synthase	1.00	8
K96243	NC006351.1	1607796	G	C	BPSS1194	non-synonymous	peptide synthase/polyketide synthase	0.98	100
K96243	NC006351.1	1607820	G	C	BPSS1194	non-synonymous	peptide synthase/polyketide synthase	0.99	99
K96243	NC006351.1	1607822	G	C	BPSS1194	synonymous	peptide synthase/polyketide synthase	0.99	99
K96243	NC006351.1	1607825	G	C	BPSS1194	synonymous	peptide synthase/polyketide synthase	0.97	102
K96243	NC006351.1	1607838	T	G	BPSS1194	non-synonymous	peptide synthase/polyketide synthase	0.99	117
K96243	NC006351.1	1607851	T	C	BPSS1194	non-synonymous	peptide synthase/polyketide synthase	0.99	111
K96243	NC006351.1	1607874	G	T	BPSS1194	non-synonymous	peptide synthase/polyketide synthase	0.97	62
K96243	NC006351.1	1607887	G	C	BPSS1194	non-synonymous	peptide synthase/polyketide synthase	1.00	58
K96243	NC006351.1	1607894	G	A	BPSS1194	synonymous	peptide synthase/polyketide synthase	0.99	72
K96243	NC006351.1	1607902	G	C	BPSS1194	non-synonymous	peptide synthase/polyketide synthase	1.00	68
K96243	NC006351.1	1607910	C	A	BPSS1194	non-synonymous	peptide synthase/polyketide synthase	0.95	57
K96243	NC006351.1	1607917	G	C	BPSS1194	non-synonymous	peptide synthase/polyketide synthase	1.00	48
K96243	NC006351.1	1607997	C	A	intergenic	N/A	N/A	0.96	49
K96243	NC006351.1	1608005	T	C	BPSS1195	stop codon destroyed	non-ribosomal peptide synthase	1.00	53
K96243	NC006351.1	1608012	G	C	BPSS1195	non-synonymous	non-ribosomal peptide synthase	0.98	55
K96243	NC006351.1	1608015	G	T	BPSS1195	non-synonymous	non-ribosomal peptide synthase	1.00	55
K96243	NC006351.1	1608017	T	A	BPSS1195	non-synonymous	non-ribosomal peptide synthase	1.00	55
K96243	NC006351.1	1608029	G	C	BPSS1195	synonymous	non-ribosomal peptide synthase	1.00	98
K96243	NC006351.1	1615675	C	T	BPSS1197	non-synonymous	BPSS1197	0.98	192
K96243	NC006351.1	1764438	G	T	intergenic	N/A	N/A	1.00	96
K96243	NC006351.1	1764448	G	T	intergenic	N/A	N/A	0.93	101
K96243	NC006351.1	2337386	A	C	BPSS1703	stop codon destroyed	hypothetical protein	1.00	188
1106a	NC_009076.1	797819	T	G	BURPS1106A_0812	non-synonymous	sensor histidine kinase	0.99	125
1026b	NC_017832.1	2020919	G	A	BP1026B_II1596	non-synonymous	type VI secretion system, VGR	0.91	297
668	NC_009074.1	3755785	G	C	BURPS668_3852	synonymous	chemotaxis protein methyltransferase	0.99	289
668	NC_009075.1	92668	CG	C	intergenic	N/A	N/A	0.91	276

### Core genome single nucleotide polymorphism (SNP) phylogeny

**Fig 1:**
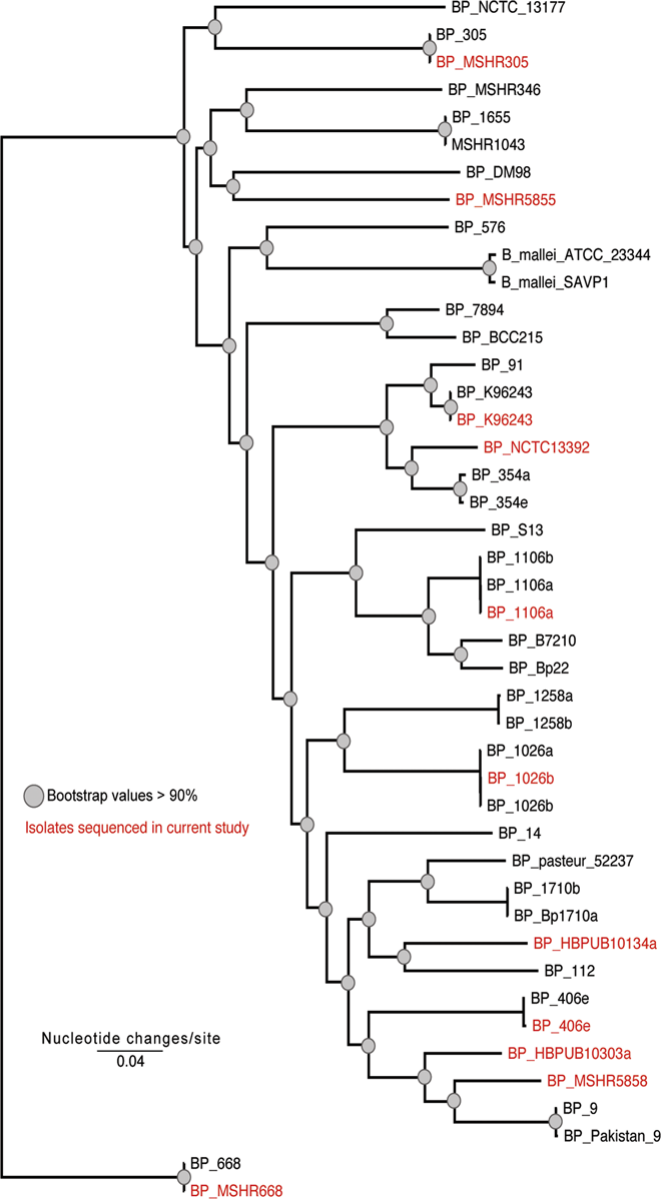
A maximum likelihood phylogeny inferred from a concatenation of ~63,000 core-genome single nucleotide polymorphisms (SNPs) identified in the eleven genomes sequenced in this study, shown in red, and a reference set of genomes (S2 Table). The tree was inferred with RAxML v8 [31, 32] using the ASC_GTRGAMMA model and 100 bootstrap replicates. Filled circles are placed at nodes where the bootstrap support values are >90%.

To phylogenetically characterize the isolates sequenced in this study, a maximum likelihood phylogeny was inferred from ~63,000 core genome SNPs (Fig. 1) identified from 44 genomes. The results demonstrate that the isolates sequenced in the current study show a broad phylogenetic history compared to previously sequenced isolates. By including phylogenetically diverse isolates in the isolate panel, local patterns of gene distribution do not bias the analysis. The retention index (RI) value of the data and maximum likelihood phylogeny demonstrated signs of homoplasy (RI = 0.62). Recombination in *B*. *pseudomallei* has been previously described [23] and homoplasy was anticipated due the recombinatorial nature of the species.

### SNP and homoplasy density

**Fig 2:**
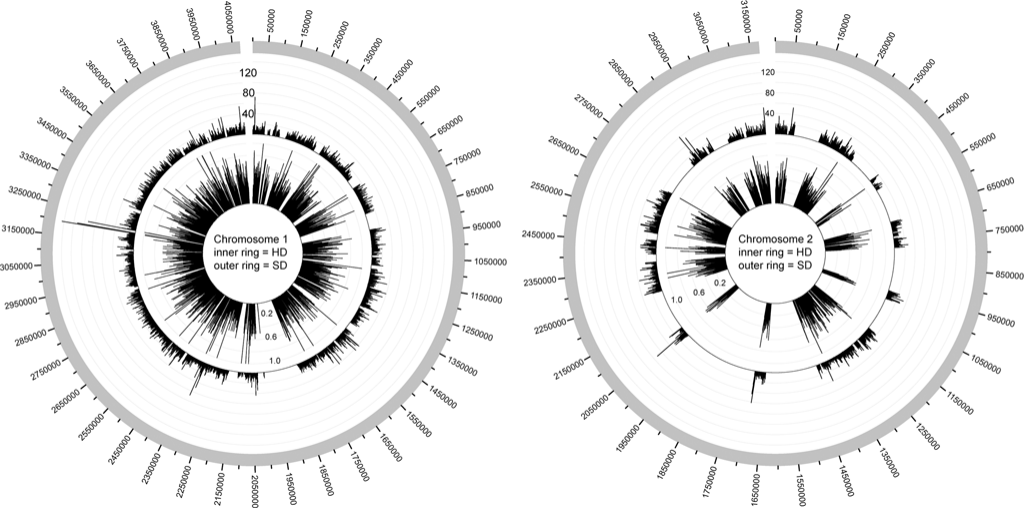
Plots of single nucleotide polymorphism (SNP) density and homoplasy density (HD), across the two chromosomes of the reference isolate, K96243 [30]. The outer ring represents the number of informative SNPs across 1-kb genomic intervals. The inner ring indicates the number of homoplasious SNPs, as determined by a retention index (RI) value <0.5 calculated by Paup [35], divided by the total number of informative SNPs over the same 1-kb genomic interval. HD and SD values were visualized with Circos [36].

The RI value of the phylogeny demonstrated the presence of homoplasy. Based on this dataset, the presence of homoplasy across the reference genome, K96243, was investigated with a SNP and homoplasy density approach. The results demonstrate that with the isolates tested, chromosome 1 of *B*. *pseudomallei* K96243 is more highly conserved than chromosome 2 (Fig. 2). Additionally, the homoplasy is distributed across both chromosomes, with no clear regions associated with specific recombination or lateral gene transfer events.

### Unique coding sequences (CDSs)

*B*. *pseudomallei* has a highly plastic genome and has the ability to acquire new genes horizontally from other microorganisms, especially as the pathogen persists in the environment. A large-scale blast score ratio (LS-BSR) analysis was performed on the 44 *B*. *pseudomallei* genomes in the phylogeny (Fig. 1) to identify any unique CDSs in the 11 isolates sequenced in the current study; the criteria for a CDS to be considered unique is that it must have a BSR value < 0.4 in all non-targeted genomes. A list of closest BLAST hits to unique CDSs not associated with either *B*. *pseudomallei* or *B*. *mallei*, based on the highest bit score, is shown in Table 3. These regions are likely associated with genomic islands horizontally transferred from related organisms [50].

**Table 3:** Annotation for unique genes identified in genomes sequenced in the current study.

Genome	closest BLAST match	closest BLAST annotation	nearest BLAST organism	protein ID (%)	query length
MSHR5858	BUPH_05469	integrase catalytic subunit	*Burkholderia phenoliruptrix*	99	99
NCTC13392	BDB_110343	hypothetical	blood disease bacterium R229	83	99
NCTC13392	BDB_110341	hypothetical	blood disease bacterium R229	82	98
10134a	YP_582472	tyrosine-based site-specific recombinase	*Cupriavidus metallidurans* CH34	64	98
10134a	YP_005995055	hypothetical	*Ralstonia solanacearum* CMR15	62	98
10134a	YP_005995053	putative integrase	*Ralstonia solanacearum* CMR15	49	98
10134a	WP_017232947	hypothetical	*Pandoraea* sp. B-6]	91	99
10134a	WP_017232948	DEAD/DEAH box helicase	*Pandoraea* sp. B-6]	91	100
10134a	WP_008918033	N-6 DNA methylase	*Burkholderia* sp. H160	78	99
10134a	WP_017232950	ATP-dependent helicase	*Pandoraea* sp. B-6]	87	99
10134a	WP_006395564	hypothetical	*Achromobacter xylosoxidans*	60	83
HBPUB10303a	YP_443256	type I restriction-modification system	*Burkholderia thailandensis* E264	78	72
HBPUB10303a	YP_443255	hypothetical	*Burkholderia thailandensis* E264	91	99
HBPUB10303a	YP_005028223	hypothetical	*Dechlorosoma suillum* PS	74	99
HBPUB10303a	WP_008248767	hypothetical	*Limnobacter* sp. MED105	51	99
HBPUB10303a	YP_006030638	helicase domain-containing protein	*Ralstonia solanacearum* Po82	79	99
MSHR5855	Bamb_2400	phage integrase family protein	*Burkholderia ambifaria* AMMD	89	96
MSHR5855	BTQ_1983	putative membrane proten	*Burkholderia thailandensis* 2002721723	99	100
MSHR5855	BTJ_373	hypothetical	*Burkholderia thailandensis* E444	99	100
MSHR5855	bglu_1g24070	hypothetical	*Burkholderia glumae* BGR1	91	99
MSHR5855	BTI_1944	hypothetical	*Burkholderia thailandensis* MSMB121	99	100
MSHR5855	BTI_1943	hypothetical	*Burkholderia thailandensis* MSMB121	78	100
MSHR5855	BTI_1942	helix-turn-helix family protein	*Burkholderia thailandensis* MSMB121	99	100
MSHR5855	Rpic12D_1056	lipoprotein releasing system	*Ralstonia pickettii* 12D	98	100

### Virulence gene profile

**Fig 3:**
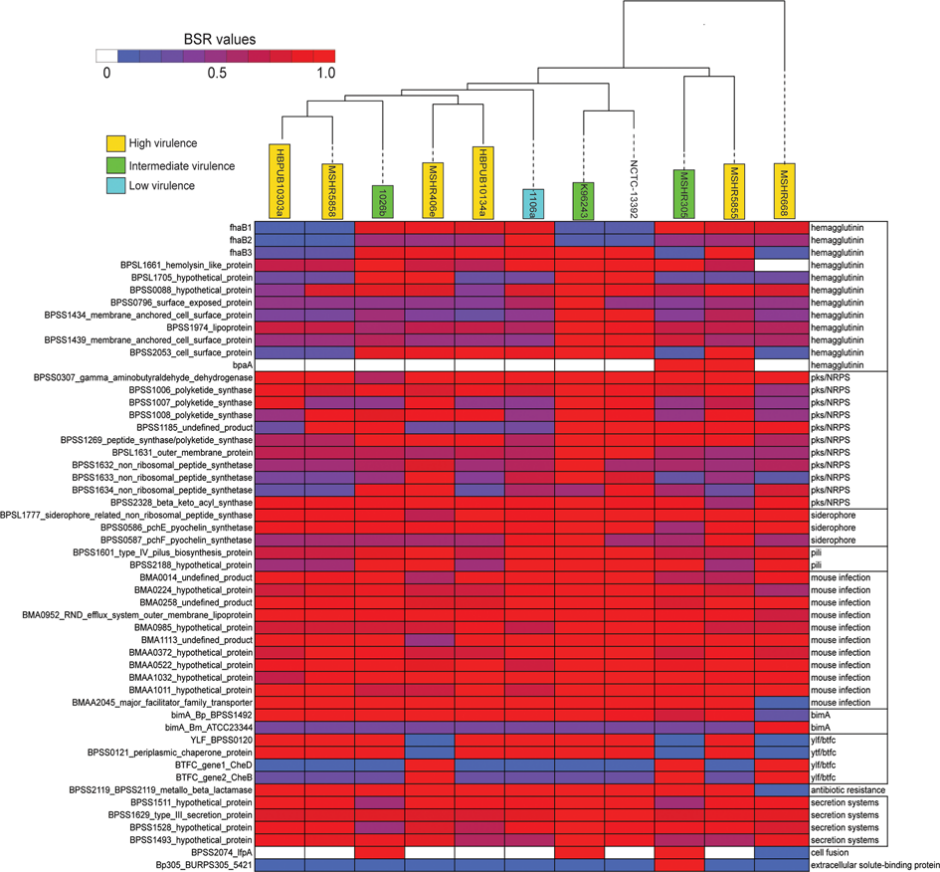
A heatmap of blast score ratio (BSR) values [44] calculated from a known set of virulence factors characterized in *B*. *pseudomallei* (S3 Table) with the large-scale blast score ratio (LS-BSR) pipeline [43]. A maximum likelihood phylogeny was inferred on a concatenation of single nucleotide polymorphisms (SNPs) and was correlated to the heatmap.

A comprehensive set of virulence-associated genes (S3 Table) was screened against the 11 genomes sequenced in this study with LS-BSR. To only compare differentially conserved regions, genes were filtered if they had a BSR value > 0.90 in all 11 genomes. The resulting variable set of genes (n = 54) was correlated to the phylogeny and LS-BSR values were visualized as a heatmap (Fig. 3). The results demonstrate that phylogenetically-distinct isolates contain a variable composition of virulence-associated genes.

Every *B*. *pseudomallei* isolate in this study contained the *B*. *pseudomallei bimA* (BimA_Bp_) allele [51], except *B*. *pseudomallei* MSHR668, which contained the alternative *B*. *mallei*-type (BimA_Bm_). The most severe clinical presentations have been associated with the co-occurrence of BimA_Bm_ with another virulence-associated gene, filamentous hemagglutinin *fhaB3* (BPSS2053 in *B*. *pseudomallei* K96243), which is linked with adhesion and heightened virulence [52, 53]. While *B*. *pseudomallei* MSHR668 is missing *fhaB3*, it does contain another *fhaB* gene (similar to *fhaB1* from *B*. *pseudomallei* MSHR305 [54]). *fhaB3* was observed in all Asian isolates in this study, which is consistent with previous work [54, 55]. Isolates sequenced in this study either contained the *Yersinia*-like fimbriae cluster (YLF) or the *B*. *thailandensis*-like flagellum and chemotaxis (BTFC) gene cluster. These genes were included in our analysis because they are suggested as being active during melioidosis.

Two isolates in this study, 1026b and MSHR305, exhibited reduced sequence homology to the T6SS-1 gene, BPSS1511. The T6SS-1 representative sequence, *icmF* gene (BPSS1511), which is required for intracellular growth of many pathogens associated with eukaryotic cells [56], showed homology, but lower sequence identity, in 1026b and MSHR305. Four isolates (MSHR5855, MSHR305, 1106a, and HBPUB10134a) exhibited reduced sequence homology for BPSS1493, a hypothetical protein associated with type VI secretion.

### Animal challenge studies

To identify differential virulence between ten of the eleven isolates sequenced in this study, BALB/c mice (seven per group) were challenged at different concentrations of inoculum (Table 4). At an average of ~10 colony forming units (CFUs) per group, four of the ten isolates killed all of the mice in the group, 5 of the isolates killed an intermediate number of mice, and one isolate (1106a) killed none of the mice (Table 4, S2 Fig, S4 Table); HBPUB10303a was treated as intermediate in terms of virulence, despite the fact that the isolate was challenged for only 14 days instead of 21 in this experiment. At a high concentration of inoculum (~12,000 CFUs), none of the mice survived when challenged with any of the ten panel isolates. This demonstrates that all of the isolates are virulent by intranasal inoculation, but there is a dose-dependent virulence response.

**Table 4:** Survival data of 10 strains injected intranasally in BALB/c mice.

Challenge Strain	Target CFU:10		Target CFU: 100		Target CFU: 1000		Target CFU: 10000	
	CFUs	%survival	CFUs	%survival	CFUs	%survival	CFUs	%survival
K96243	13(+/-0)	57	170(+/-21)	14	1581(+/-212)	0	11940(+/-1485)	0
MSHR406e	13(+/-0)	0	135(+/-4)	0	1365(+/-106)	0	12840(+/-636)	0
1026b	12(+/-0)	57	127(+/-11)	0	1215(+/-106)	0	11355(+/-785)	0
1106a	12(+/-0)	100	128(+/-23)	71	1220(+/-112)	29	12090(+/-2121)	0
MSHR305	16(+/-1)	29	149(+/-15)	0	1524(+/-21)	0	14100(+/-2121)	0
MSHR668	14(+/-0)	0	122(+/-2)	0	1095(+/-8)	0	12945(+/-912)	0
MSHR5855	16(+/-1)	0	185(+/-48)	0	1575(+/-136)	0	14550(+/-933)	0
MSHR5858	14(+/-2)	86	154(+/-8)	14	1485(+/-119)	0	15750(+/-2630)	0
HBPUB10303a	6(+/-0)	71	59(+/-1)	14	537(+/-38)	0	5280(+/-424)	0
HBPUB10134a	16	0	163	0	1779	0	18810	0

### Genotype and phenotype correlations

Differences were observed in both the virulence gene profile and the animal challenge studies. To identify if any CDSs were associated with differential virulence, a combined LS-BSR/QIIME analysis was performed. A Kruskal-Wallis test [45] demonstrated that numerous CDSs were significantly (false detection rate adjusted (FDR) p<0.05) differentially conserved between groups (Table 5); three of these CDSs (BPSS0771, BPSS1185, BPSS1269) have previously been associated with virulence (Table 5). Additionally, an association was made between core genome SNPs and differential virulence. Forty SNPs were only identified in high virulence isolates (Table 6), which could be due to descent and subsequent loss by intermediate and low virulence isolates, but may also be associated with convergent evolution and virulence (Fig. 3). By randomly assigning genomes to high and low virulence groups, an average of 31 correlated SNPs were identified over ten iterations. This demonstrates that with small sample sets, identified correlations would definitely need to be corroborated with functional characterization.

**Table 5:** Correlations of LS-BSR values with observed differential virulence in BALB/c mice.

Accession	Annotation	BSR average (high)*	BSR average (intermediate)*	BSR average (low)*	FDR p-value
BPSS1185	undefined product	73.5	87.2	14	0.0000
BPSL2990	histone H1-like protein	100	71	41	0.0003
BPSL0016	general secretory pathway protein L	100	99.8	51	0.0022
BDL_4286	response regulator	97.25	87.2	49	0.0082
BPSS1308	isoaspartyl peptidase	100	99.6	54	0.0082
BPSL0859	N-acetylmuramoyl-L-alanine amidase	100	100	55	0.0106
BPSS0771	hypothetical protein	98	81.6	50	0.0147
BPSS1269	peptide_synthase/polyketide_synthase	87.75	99.2	54	0.0324
BPSS1212	hypothetical protein	99.75	78.4	54	0.0454

*High, intermediate and low virulence determined by intranasal challenge at ~10 colony forming units.

**Table 6:** Single nucleotide polymorphisms (SNPs) unique to high virulence isolates.

Chrom	Coordinate	K96243 call	query call	refAA	derivedAA	locus tag	Annotation
NC_006350.1	220073	C	T	R	Q	BPSL0211	lipid A biosynthesis lauroyl acyltransferase
NC_006350.1	220163	C	T	R	Q	BPSL0211	lipid A biosynthesis lauroyl acyltransferase
NC_006350.1	240744	T	C	M	V	BPSL0230	fliF; flagellar MS-ring protein
NC_006350.1	534205	G	T	A	D	BPSL0492	hypothetical protein
NC_006350.1	554241	T	C	A	A	BPSL0504	rpoH; RNA polymerase factor sigma-32
NC_006350.1	1839157	C	G	N/A	N/A	intergenic	N/A
NC_006350.1	1839166	G	A	N/A	N/A	intergenic	N/A
NC_006350.1	1839172	T	C	N/A	N/A	intergenic	N/A
NC_006350.1	1839197	A	C	N/A	N/A	intergenic	N/A
NC_006350.1	1839256	A	G	N/A	N/A	intergenic	N/A
NC_006350.1	1839334	C	T	N/A	N/A	intergenic	N/A
NC_006350.1	1839988	T	C	K	R	BPSL1583	hypothetical protein
NC_006350.1	1840050	G	A	A	A	BPSL1583	hypothetical protein
NC_006350.1	2402720	T	C	N/A	N/A	intergenic	N/A
NC_006350.1	2440328	A	G	D	D	BPSL2041	hypothetical protein
NC_006350.1	2553415	C	G	A	A	BPSL2126	transport-related, membrane protein
NC_006350.1	2555149	G	A	N/A	N/A	intergenic	N/A
NC_006350.1	2555151	C	T	N/A	N/A	intergenic	N/A
NC_006350.1	2820597	A	G	I	T	BPSL2334	hypothetical protein
NC_006350.1	2847891	T	C	N/A	N/A	intergenic	N/A
NC_006350.1	3021077	A	C	N/A	N/A	intergenic	N/A
NC_006350.1	3403668	G	T	T	T	BPSL2842	FAD-binding oxidase
NC_006350.1	3654489	C	G	N/A	N/A	intergenic	N/A
NC_006350.1	3924581	G	A	H	H	BPSL3305	cheW; chemotaxis protein
NC_006350.1	4073158	T	C	E	G	BPSL3430	glutamine amidotransferase
NC_006351.1	702758	G	A	G	S	BPSS0515	hypothetical protein
NC_006351.1	1236140	T	G	P	P	BPSS0936	hypothetical protein
NC_006351.1	1269935	T	C	N/A	N/A	intergenic	N/A
NC_006351.1	1398575	C	T	P	P	BPSS1026	hypothetical protein
NC_006351.1	1398581	A	G	*	W	BPSS1026	hypothetical protein
NC_006351.1	1917766	T	C	N/A	N/A	intergenic	N/A
NC_006351.1	2455301	T	G	S	A	BPSS1795	hypothetical protein
NC_006351.1	2514965	G	A	N/A	N/A	intergenic	N/A
NC_006351.1	2695450	C	T	N/A	N/A	intergenic	N/A
NC_006351.1	3000485	G	A	N/A	N/A	intergenic	N/A
NC_006351.1	3042744	A	G	D	D	BPSS2265	monooxygenase
NC_006351.1	3097464	G	A	N/A	N/A	intergenic	N/A
NC_006351.1	3097471	C	T	N/A	N/A	intergenic	N/A
NC_006351.1	3097776	C	A	N/A	N/A	intergenic	N/A
NC_006351.1	3160929	G	A	N/A	N/A	intergenic	N/A

## Discussion

*Burkholderia pseudomallei* is an important pathogen as both the causative agent of melioidosis and as a potential biothreat agent. In the development of medical countermeasures against melioidosis, a panel of clinically relevant isolates have been identified [7] for challenge studies under the FDA Animal Rule [8]. In this study, we sequenced all 6 of these isolates as well as 5 additional isolates associated with human inhalational melioidosis. A comparative genomics approach was employed to understand the genetic composition of each genome and the distribution of genetic elements between genomes. These results were correlated with animal survival data to determine if phenotype/genotype correlations could be identified.

Ten of the 11 isolates were passed through a BALB/c mouse model in groups of seven mice per isolate. Differential virulence was observed between isolates, with MSHR668 demonstrating the highest virulence (S2 Fig, Table 2), based on time to death. An attempt was made to correlate both the distribution of coding sequences (CDSs), based on large-scale blast score ratio (LS-BSR) values, and single nucleotide polymorphisms (SNPs), with differential virulence. Three CDSs previously associated with virulence were differentially conserved between disease severity groups (Table 4). Additionally, SNPs were identified that were only present in high-virulence isolates (Table 6). While the limited number of isolates tested in this study precludes definitive correlations between genotype and phenotype, differentially conserved CDSs and/or SNPs may inform larger-scale targeted functional studies, which may help to better understand the pathogenesis of *B*. *pseudomallei*, and subsequently, may improve human health.

A maximum likelihood phylogeny inferred from a concatenation of ~60,000 core-genome SNPs demonstrated that the eleven isolates sequenced in the current study represent broad phylogenetic diversity. The retention index (RI) value, which provides a representation of the homoplasy in the dataset, demonstrated signs of homoplasy, which can confound accurate phylogenetic reconstruction. Plotting the observed homoplasy density (HD) across both chromosomes of *B*. *pseudomallei* K96243 demonstrated that the homoplasy was evenly distributed, with no isolated regions of recombination in the core genome. Although this underlying homoplasy may confound phylogenetic relationships, especially in deeply branching nodes, the phylogeny still demonstrates the overall diversity of the eleven isolates sequenced in the current study.

Differences in the distribution of virulence-associated genes were observed based a LS-BSR analysis. One clear difference was the presence of the *B*. *mallei bimA* (*BimA*_Bm_) allele in MSHR668 and the *B*. *pseudomallei* version (*BimA*_Bp_) in all other isolates (Fig. 3). In previous studies, 12% of Australian isolates contained *BimA*_Bm_ [55, 57], although both versions appear to perform actin-based motility effectively. An association between neurological melioidosis and strains with *BimA*_Bm_ was recently reported [55]. Severe clinical presentations have been associated with the co-occurrence of *BimA*_Bm_ and the hemagglutinin, *fhaB3*. The lack of *fhaB3* in isolates exhibiting *BimA*_Bm_ was correlated with cutaneous melioidosis without sepsis [55]. Testing isolates with varied distributions of these virulence components will help corroborate these associations.

The Inv/Mxi-Spa-like type III secretion system (T3SS-3) [58] is essential for the survival of *B*. *pseudomallei* in the host [59, 60] and closely resembles secretion systems found in other animal pathogens (*Salmonella* spp. and *Shigella* spp.). *B*. *pseudomallei* isolates 1026b and HBPUB10134a appear to have reduced homology for BPSS1528, which is described as a (HNS-like regulatory) hypothetical protein in the T3SS-3 system. Several proteins act together to form a pore that becomes bound to the host membrane, thus facilitating the delivery of effector proteins [61, 62]. This system is also likely involved in defenses against autophagy by transporting the BopA effector [63, 64]. In this study, we observed sequence homology variation among many of the isolates in the gene, BPSS1629, from the T3SS-2 cluster.

One of the most dramatic differences observed between isolates was from representative genes in the *Yersinia*-like fimbriae (YLF) gene cluster and the BTFC gene cluster. This division is mutually exclusive [54, 55] and it is unclear whether one cluster confers enhanced virulence over the other and no correlations have been identified between gene cluster and disease severity [55]. While YLF genes are generally associated with isolates from Thailand [55], we found no geographical correlation in the small sample set that we analyzed in the current study (Fig. 3).

The FDA Animal Rule was set up to identify a set of relevant isolates that could be used in lieu of human clinical trials in the development of effective medical countermeasures against human disease, including melioidosis. The data presented in this study will provide a genomic background to better understand virulence in *B*. *pseudomallei* and may also help in the development of more effective medical countermeasures.

## Supporting Information

S1 Dataset The complete LS-BSR matrix for all coding regions in each genome investigated. (BZ2)

S2 Dataset A NASP (http://tgennorth.github.io/NASP/) matrix containing all SNPs from non-duplicated regions from all genomes queried. (BZ2)

S1 Fig Synteny dot plots between finished genomes available in GenBank and draft genomes generated in this study from re-sequencing studies. Dot plots were generated using the mummerplot method in MUMmer.
(TIF)

S2 Fig A Kaplan-Meier curve of survival probabilities based on the BALB/c mice challenge studies conducted in the current study. The survival probabilities were calculated using the ‘survival’ package in R [12].
(TIF)

S1 Table Sequencing information for isolates sequenced in the current study. (PDF)

S2 Table Accession information for reference genomes. (PDF)

S3 Table Virulence associated genes in the current study. (PDF)

S4 Table Survival information over the course of BALB/c challenge studies for all strains challenged. (PDF)
