# Supplementary figures and images for: Genomic Characterization of Burkholderia pseudomallei Isolates Selected for Medical Countermeasures Testing: Comparative Genomics Associated with Differential Virulence
Source: PLoS One. 2015 Mar 24;10(3):e0121052. doi: 10.1371/journal.pone.0121052 (PMC4372212; doi:10.1371/journal.pone.0121052)

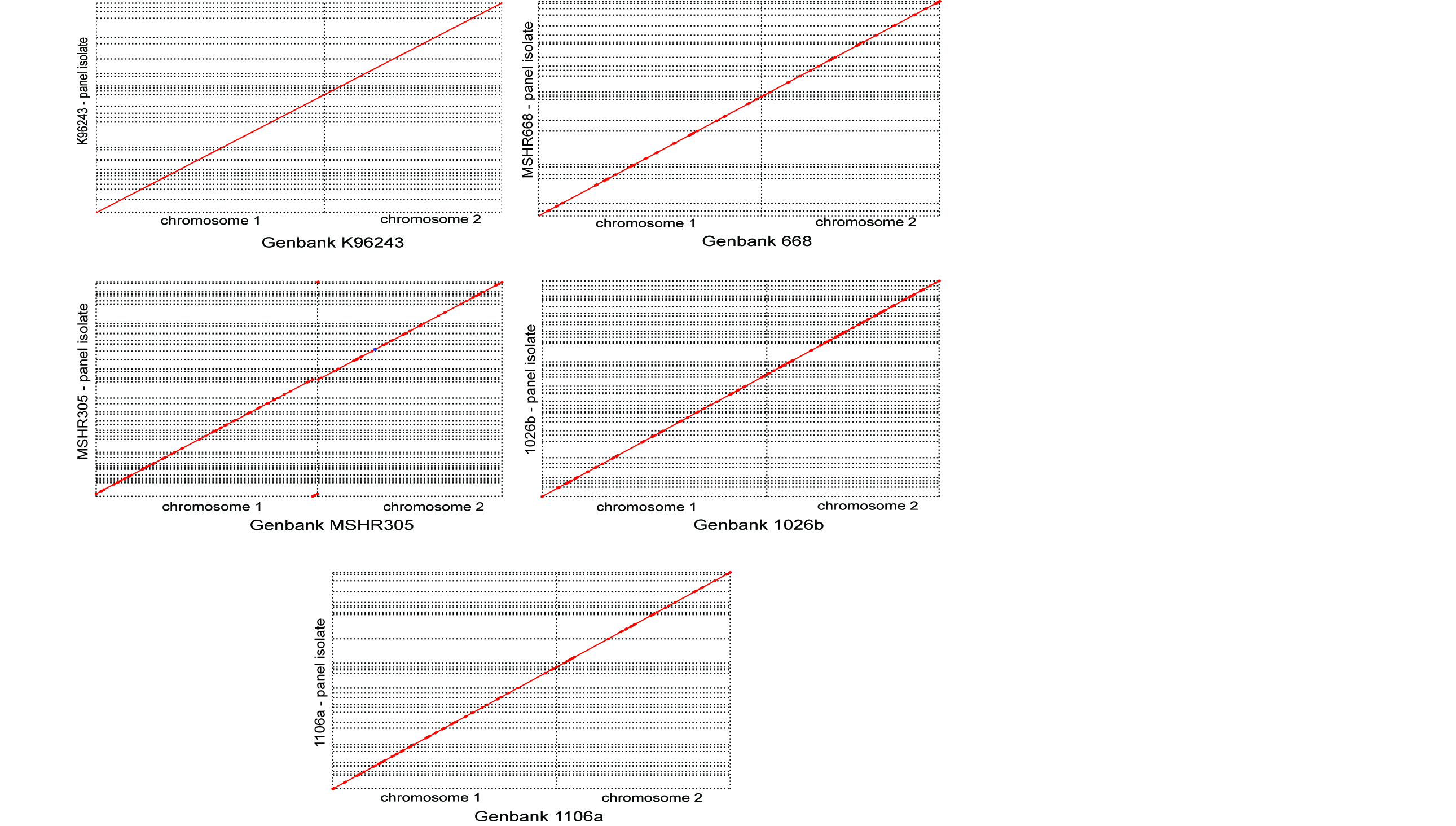

Supplement: S1 Fig — Dot plots were generated using the mummerplot method in MUMmer. (TIF) [file pone.0121052.s003.tif]

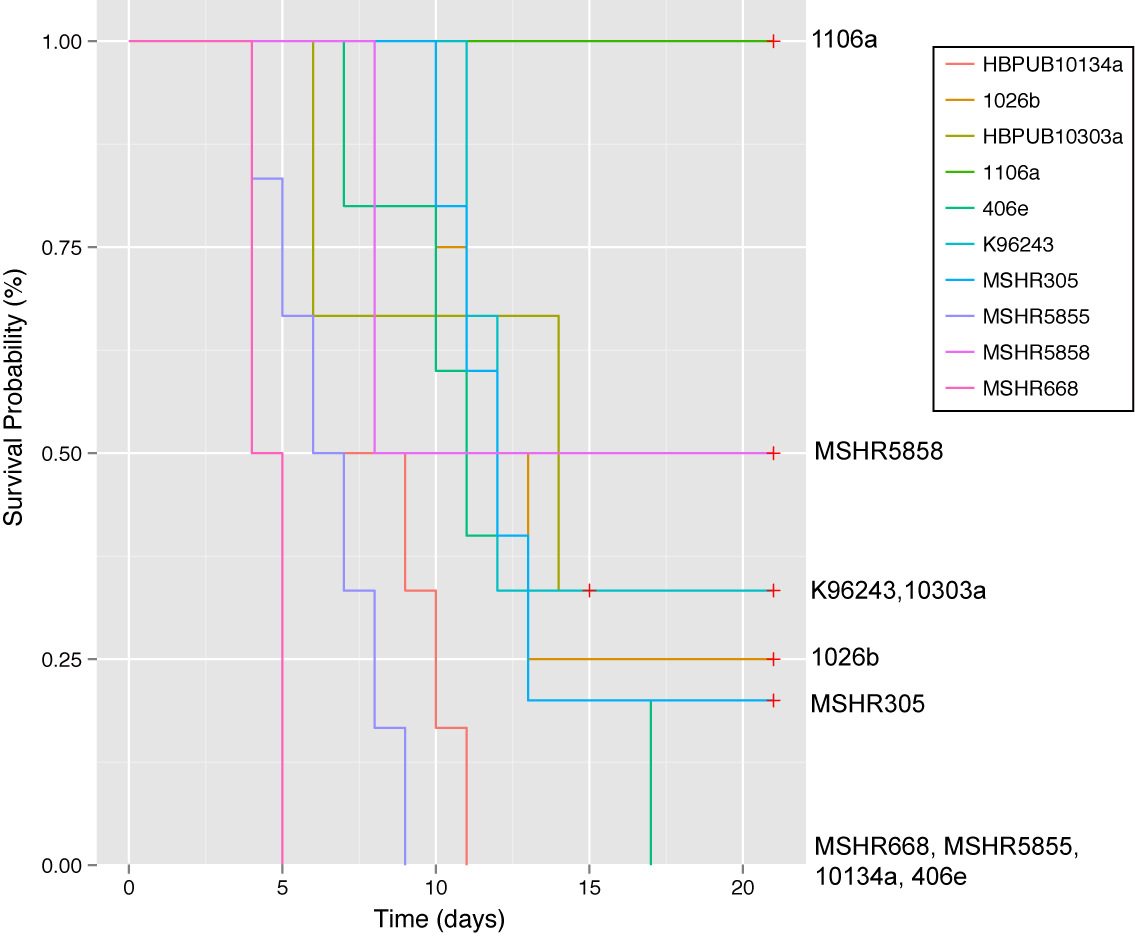

Supplement: S2 Fig — The survival probabilities were calculated using the ‘survival’ package in R [12]. (TIF) [file pone.0121052.s004.tif]
