# Supplementary material for: Genomic Characterization of Burkholderia pseudomallei Isolates Selected for Medical Countermeasures Testing: Comparative Genomics Associated with Differential Virulence
Source: PLoS One. 2015 Mar 24;10(3):e0121052. doi: 10.1371/journal.pone.0121052 (PMC4372212; doi:10.1371/journal.pone.0121052)
Supplement: S1 Table — (PDF) [file pone.0121052.s005.pdf]

S1 Table. Sequencing information for isolates sequenced in the current study

Isolate	#contigs	N50	Assembly size	Average coverage depth	Assembly type	Reference genome	MLST							
							ace	gltB	gmhD	lepA	lipA	narK	ndh	ST
1106a	36	354834	7092838	251x	reference guided + de novo	NC_009076.1,NC_009078.1	3	4	11	3	5	4	6	70
K96243	31	624884	7255990	129x	reference guided + de novo	NC_006350.1,NC_006351.1	1	1	13	1	1	1	1	10
MSHR305	61	240788	7433229	256x	reference guided + de novo	AAYX00000000	1	7	14	7	1	12	11	36
MSHR668	25	437234	7045801	170x	reference guided + de novo	NC_009075.1,NC_009074.1	1	15	13	2	1	22	1	129
406e	115	175239	7235897	285x	de novo	N/A	3	1	3	1	1	4	1	211
NCTC 13392	48	418318	7164815	228x	reference guided + de novo	NC_006350.1,NC_006351.1	1	2	13	1	1	1	1	23
1026b	53	204505	7241494	169x	reference guided + de novo	NC_017831.1,NC_017832.1	3	4	12	1	1	4	1	102
MSHR5855	159	196960	7289430	177x	de novo	N/A	1	1	3	4	30	2	30	553
MSHR5858	128	115461	7257556	141x	de novo	N/A	1	1	4	1	1	29	1	562
HBPUB10134a	124	121543	7331046	160x	de novo	N/A	1	2	3	1	1	4	1	228
HBPUB10303a	116	131427	7339656	173x	de novo	N/A	3	1	2	1	1	4	1	48
