# Supplementary material for: Genomic Characterization of Burkholderia pseudomallei Isolates Selected for Medical Countermeasures Testing: Comparative Genomics Associated with Differential Virulence
Source: PLoS One. 2015 Mar 24;10(3):e0121052. doi: 10.1371/journal.pone.0121052 (PMC4372212; doi:10.1371/journal.pone.0121052)
Supplement: S2 Table — (PDF) [file pone.0121052.s006.pdf]

**S2 Table.** Accession information for reference genomes

| Genome | Accession |
|---|---|
| 1026a | AHJA00000000 |
| 1026b | NC_017831.1,NC_017832.1 |
| 1106a | NC_009076.1,NC_009078.1 |
| 1106b | AAMB00000000 |
| 112 | ABBP00000000 |
| 1258a | AHJB00000000 |
| 1258b | AHJC00000000 |
| 14 | ABBJ00000000 |
| 1655 | AAHR00000000 |
| 1710b | NC_007434.1,CP000125.1 |
| 305 | AAYX00000000 |
| 354a | AGVS00000000 |
| 354e | AHJD00000000 |
| 406e | AAMM00000000 |
| 576 | ACCE00000000 |
| 668 | NC_009075.1,NC_009074.1 |
| 7894 | ABBO00000000 |
| 91 | ABBK00000000 |
| 9 | ABBL00000000 |
| B7210 | ABBN00000000 |
| BCC215 | ABBR00000000 |
| Bp1710a | AAHS00000000 |
| Bp22 | AFBJ00000000 |
| DM98 | ABBI00000000 |
| K96243 | NC_006350.1,NC_006351.1 |
| MSHR346 | NC_012695.1,ACOJ00000000 |
| NCTC-13177 | ABBQ00000000 |
| Pakistan 9 | ACKA00000000 |
| pasteur 52237 | AAHV00000000 |
| S13 | AAHW00000000 |
| MSHR1043 | AOGU00000000 |
| *B. mallei* 23344 | NC_006348.1,NC_006349.2 |
| *B. mallei* SAVP1 | CP000526.1,CP000525.1 |
