# Supplementary material for: Genomic Characterization of Burkholderia pseudomallei Isolates Selected for Medical Countermeasures Testing: Comparative Genomics Associated with Differential Virulence
Source: PLoS One. 2015 Mar 24;10(3):e0121052. doi: 10.1371/journal.pone.0121052 (PMC4372212; doi:10.1371/journal.pone.0121052)
Supplement: S3 Table — (PDF) [file pone.0121052.s007.pdf]

S3 Table. Virulence associated genes screened in the current study

locus tag	annotation	
BBK_5271	BTFC (gene 1)	Tuanyok *et al.* 2007
BDL_981	fhaB1_MSHR305	Tuanyok *et al.* 2008
BMA0014	hypothetical protein	Nierman *et al.* 2004, Kim et al. 2005
BMA0224	hypothetical protein	Nierman *et al.* 2004, Kim et al. 2005
BMA0258	hypothetical protein	Nierman *et al.* 2004, Kim et al. 2005
BMA0357	conserved domain protein	Nierman *et al.* 2004, Kim et al. 2005
BMA0952	RND efflux system, outer membrane lipoprotein, NodT family	Nierman *et al.* 2004, Kim et al. 2005
BMA0985	hypothetical protein	Nierman *et al.* 2004, Kim et al. 2005
BMA0989	hypothetical protein	Nierman *et al.* 2004, Kim et al. 2005
BMA1038	penicillin amidase, putative	Nierman *et al.* 2004, Kim et al. 2005
BMA1113	conserved hypothetical protein	Nierman *et al.* 2004, Kim et al. 2005
BMA1123	peptide synthetase, putative	Nierman *et al.* 2004, Kim et al. 2005
BMA1754	CoA transferase, CAIB/BAIF family	Nierman *et al.* 2004, Kim et al. 2005
BMA1944	conserved hypothetical protein	Nierman *et al.* 2004, Kim et al. 2005
BMA2157	conserved hypothetical protein	Nierman *et al.* 2004, Kim et al. 2005
BMA2247	hypothetical protein	Nierman *et al.* 2004, Kim et al. 2005
BMA2576	phenylacetic acid degradation protein PaaD	Nierman *et al.* 2004, Kim et al. 2005
BMA2880	3-oxoacyl-(acyl-carrier-protein) synthase III, putative	Nierman *et al.* 2004, Kim et al. 2005
BMA3058	carotenoid 9,10-9',10' cleavage dioxygenase, putative	Nierman *et al.* 2004, Kim et al. 2005
BMA3068	hypothetical protein	Nierman *et al.* 2004, Kim et al. 2005
BMA3158	AMP-binding domain protein	Nierman *et al.* 2004, Kim et al. 2005
BMA3224	conserved hypothetical protein	Nierman *et al.* 2004, Kim et al. 2005
BMAA0023	cytochrome P450-related protein	Nierman *et al.* 2004, Kim et al. 2005
BMAA0059	conserved hypothetical protein	Nierman *et al.* 2004, Kim et al. 2005
BMAA0073	hypothetical protein	Nierman *et al.* 2004, Kim et al. 2005
BMAA0098	hypothetical protein	Nierman *et al.* 2004, Kim et al. 2005
BMAA0372	hypothetical protein	Nierman *et al.* 2004, Kim et al. 2005
BMAA0522	hypothetical protein	Nierman *et al.* 2004, Kim et al. 2005
BMAA0594	hypothetical protein	Nierman *et al.* 2004, Kim et al. 2005
BMAA0616	hypothetical protein	Nierman *et al.* 2004, Kim et al. 2005
BMAA0618	hypothetical protein	Nierman *et al.* 2004, Kim et al. 2005
BMAA0619	transcriptional regulator, MarR family	Nierman *et al.* 2004, Kim et al. 2005
BMAA0628	hypothetical protein	Nierman *et al.* 2004, Kim et al. 2005
BMAA0630	aldehyde dehydrogenase (NADP) family protein	Nierman *et al.* 2004, Kim et al. 2005
BMAA0646	hypothetical protein	Nierman *et al.* 2004, Kim et al. 2005
BMAA0651	H-NS histone family protein	Nierman *et al.* 2004, Kim et al. 2005
BMAA0664	EAL/GGDEF domain protein	Nierman *et al.* 2004, Kim et al. 2005
BMAA0673	hypothetical protein	Nierman *et al.* 2004, Kim et al. 2005
BMAA0689	hypothetical protein	Nierman *et al.* 2004, Kim et al. 2005
BMAA0703	DNA-binding response regulator, LuxR family	Nierman *et al.* 2004, Kim et al. 2005
BMAA0728	hypothetical protein	Nierman *et al.* 2004, Kim et al. 2005
BMAA0749	bimA_Bm_ATCC23344	Nierman *et al.* 2004, Kim et al. 2005
BMAA0751	N-acetylmuramoyl-L-alanine amidase domain protein	Nierman *et al.* 2004, Kim et al. 2005
BMAA0752	hypothetical protein	Nierman *et al.* 2004, Kim et al. 2005
BMAA0798	mulitcopper oxidase domain protein	Nierman *et al.* 2004, Kim et al. 2005
BMAA0841	conserved hypothetical protein, degenerate	Nierman *et al.* 2004, Kim et al. 2005
BMAA0955	hypothetical protein	Nierman *et al.* 2004, Kim et al. 2005
BMAA0957	conserved hypothetical protein	Nierman *et al.* 2004, Kim et al. 2005
BMAA1011	hypothetical protein	Nierman *et al.* 2004, Kim et al. 2005
BMAA1032	hypothetical protein	Nierman *et al.* 2004, Kim et al. 2005
BMAA1133	transcriptional regulator, AraC family	Nierman *et al.* 2004, Kim et al. 2005
BMAA1149	hypothetical protein	Nierman *et al.* 2004, Kim et al. 2005
BMAA1151	conserved hypothetical protein	Nierman *et al.* 2004, Kim et al. 2005
BMAA1162	hypothetical protein	Nierman *et al.* 2004, Kim et al. 2005
BMAA1164	membrane protein, putative	Nierman *et al.* 2004, Kim et al. 2005
BMAA1184	conserved hypothetical protein	Nierman *et al.* 2004, Kim et al. 2005
BMAA1202	polyketide synthase, putative, degenerate	Nierman *et al.* 2004, Kim et al. 2005
BMAA1211	hypothetical protein	Nierman *et al.* 2004, Kim et al. 2005
BMAA1248	serine metalloprotease MrpA	Nierman *et al.* 2004, Kim et al. 2005
BMAA1252	hypothetical protein	Nierman *et al.* 2004, Kim et al. 2005
BMAA1390	conserved hypothetical protein	Nierman *et al.* 2004, Kim et al. 2005
BMAA1395	cyclic nucleotide-binding domain protein	Nierman *et al.* 2004, Kim et al. 2005
BMAA1434	hypothetical protein	Nierman *et al.* 2004, Kim et al. 2005
BMAA1486	O-methyltransferase family protein	Nierman *et al.* 2004, Kim et al. 2005
BMAA1488	hypothetical protein	Nierman *et al.* 2004, Kim et al. 2005
BMAA1498	O-antigen acetylase, putative	Nierman *et al.* 2004, Kim et al. 2005
BMAA1568	serine protease, kumamolysin	Nierman *et al.* 2004, Kim et al. 2005
BMAA1617	hrp protein, putative	Nierman *et al.* 2004, Kim et al. 2005
BMAA1619	hypothetical protein	Nierman *et al.* 2004, Kim et al. 2005
BMAA1690	luciferase-like monooxygenase	Nierman *et al.* 2004, Kim et al. 2005
BMAA1757	hemolysin activator protein, HlyB family	Nierman *et al.* 2004, Kim et al. 2005
BMAA1841	glyoxalase family protein	Nierman *et al.* 2004, Kim et al. 2005
BMAA1842	hypothetical protein	Nierman *et al.* 2004, Kim et al. 2005
BMAA1865	conserved hypothetical protein	Nierman *et al.* 2004, Kim et al. 2005
BMAA1875	hypothetical protein	Nierman *et al.* 2004, Kim et al. 2005
BMAA1895	conserved domain protein	Nierman *et al.* 2004, Kim et al. 2005
BMAA1902	conserved hypothetical protein	Nierman *et al.* 2004, Kim et al. 2005
BMAA1912	conserved hypothetical protein	Nierman *et al.* 2004, Kim et al. 2005
BMAA1925	hypothetical protein	Nierman *et al.* 2004, Kim et al. 2005
BMAA1970	conserved hypothetical protein	Nierman *et al.* 2004, Kim et al. 2005
BMAA1973	conserved hypothetical protein	Nierman *et al.* 2004, Kim et al. 2005
BMAA1983	hypothetical protein	Nierman *et al.* 2004, Kim et al. 2005
BMAA1986	ADP-heptose--LPS heptosyltransferase II, putative	Nierman *et al.* 2004, Kim et al. 2005
BMAA1987	glycosyl transferase, group 2 family protein	Nierman *et al.* 2004, Kim et al. 2005
BMAA1995	conserved domain protein	Nierman *et al.* 2004, Kim et al. 2005
BMAA2006	flavin reductase domain protein	Nierman *et al.* 2004, Kim et al. 2005
BMAA2014	hypothetical protein	Nierman *et al.* 2004, Kim et al. 2005
BMAA2016	hypothetical protein	Nierman *et al.* 2004, Kim et al. 2005
BMAA2045	major facilitator family transporter	Nierman *et al.* 2004, Kim et al. 2005
BMAA2047	molybdopterin oxidoreductase family protein	Nierman *et al.* 2004, Kim et al. 2005
BPSL0071	putative catalase	Holden *et al*. 2004
BPSL0338	PLC-2, non-hemolytic phospholipase C precursor (EC:3.1.4.3) - secreted factor	Holden *et al*. 2004
BPSL0374	Ambler class B b -lactamase	Holden *et al*. 2004
BPSL0624	*P. aeruginosa* LasA elastase	Holden *et al*. 2004
BPSL0782	pilA; putative type 4 fimbrial pilin protein; B. pseudomallei binds to aGM1-GM2 receptor complex; P. aeruginosa does this via type IV pili	Holden *et al*. 2004
BPSL0808	homologue of the *P. aeruginosa* MucD Ser protease	Holden *et al*. 2004
BPSL0814	BpeA (K96243)	Holden *et al*. 2004
BPSL0815	BpeB (K96243)	Holden *et al*. 2004
BPSL0816	AcrAB-OprM multi-drug efflux pump	Holden *et al*. 2004
BPSL0880	*sodB*	Holden *et al*. 2004
BPSL1001	*sodC*	Holden *et al*. 2004
BPSL1007	Type I fimbriae 1	Holden *et al*. 2004
BPSL1008	Type I fimbriae 1	Holden *et al*. 2004
BPSL1036	*ompR*	Holden *et al*. 2004
BPSL1108	hemagglutinin-related protein	Holden *et al*. 2004
BPSL1471	*pmrF*	Holden *et al*. 2004
BPSL1505	*rpoS*	Holden *et al*. 2004
BPSL1505	RpoS- induction of MNGC; RNA polymerase, sigma 38 subunit (K96243)	Holden *et al*. 2004
BPSL1561	Ambler class B b -lactamase	Holden *et al*. 2004
BPSL1626	Type I fimbriae 2	Holden *et al*. 2004
BPSL1627	Type I fimbriae 2	Holden *et al*. 2004
BPSL1628	Type I fimbriae 2	Holden *et al*. 2004
BPSL1629	Type I fimbriae 2	Holden *et al*. 2004
BPSL1631	Hep_Hag family hemagglutinin-like protein	Holden *et al*. 2004
BPSL1633	MrgR (K96243)	Holden *et al*. 2004
BPSL1634	MrgS (K96243)	Holden *et al*. 2004
BPSL1661	putative hemagglutinin/hemolysin-related protein	Holden *et al*. 2004
BPSL1705	Hep_Hag family hemagglutinin-like protein	Holden *et al*. 2004
BPSL1710	Putative nonribosomal peptide synthase (NRPS) cluster 1	Holden *et al*. 2004
BPSL1711	Putative nonribosomal peptide synthase (NRPS) cluster 1	Holden *et al*. 2004
BPSL1712	Putative nonribosomal peptide synthase (NRPS) cluster 1	Holden *et al*. 2004
BPSL1713	Putative nonribosomal peptide synthase (NRPS) cluster 1	Holden *et al*. 2004
BPSL1714	Putative nonribosomal peptide synthase (NRPS) cluster 1	Holden *et al*. 2004
BPSL1715	Putative nonribosomal peptide synthase (NRPS) cluster 1	Holden *et al*. 2004
BPSL1716	Putative nonribosomal peptide synthase (NRPS) cluster 1	Holden *et al*. 2004
BPSL1717	Putative nonribosomal peptide synthase (NRPS) cluster 1	Holden *et al*. 2004
BPSL1718	Putative nonribosomal peptide synthase (NRPS) cluster 1	Holden *et al*. 2004
BPSL1719	Putative nonribosomal peptide synthase (NRPS) cluster 1	Holden *et al*. 2004
BPSL1720	Putative nonribosomal peptide synthase (NRPS) cluster 1	Holden *et al*. 2004
BPSL1721	Putative nonribosomal peptide synthase (NRPS) cluster 1	Holden *et al*. 2004
BPSL1722	Putative nonribosomal peptide synthase (NRPS) cluster 1	Holden *et al*. 2004
BPSL1723	Putative nonribosomal peptide synthase (NRPS) cluster 1	Holden *et al*. 2004
BPSL1724	Putative nonribosomal peptide synthase (NRPS) cluster 1	Holden *et al*. 2004
BPSL1725	Putative nonribosomal peptide synthase (NRPS) cluster 1	Holden *et al*. 2004
BPSL1726	Putative nonribosomal peptide synthase (NRPS) cluster 1	Holden *et al*. 2004
BPSL1727	Putative nonribosomal peptide synthase (NRPS) cluster 1	Holden *et al*. 2004
BPSL1774	Putative hydroxamate siderophore biosynthesis cluster. Other genes possibly involved in the production of the siderophore	Holden *et al*. 2004
BPSL1775	Putative hydroxamate siderophore biosynthesis cluster. Other genes possibly involved in the production of the siderophore	Holden *et al*. 2004
BPSL1776	Putative hydroxamate siderophore biosynthesis cluster. Other genes possibly involved in the production of the siderophore	Holden *et al*. 2004
BPSL1777	Putative hydroxamate siderophore biosynthesis cluster. Other genes possibly involved in the production of the siderophore	Holden *et al*. 2004
BPSL1778	Putative hydroxamate siderophore biosynthesis cluster. Other genes possibly involved in the production of the siderophore	Holden *et al*. 2004
BPSL1779	Putative hydroxamate siderophore biosynthesis cluster. Other genes possibly involved in the production of the siderophore	Holden *et al*. 2004
BPSL1799	Type I fimbriae 3	Holden *et al*. 2004
BPSL1800	Type I fimbriae 3	Holden *et al*. 2004
BPSL1801	Type I fimbriae 3	Holden *et al*. 2004
BPSL1802	AmrAB multi-drug efflux pump	Holden *et al*. 2004
BPSL1803	AmrAB multi-drug efflux pump	Holden *et al*. 2004
BPSL1804	AmrAB multi-drug efflux pump	Holden *et al*. 2004
BPSL1813	*tad*-like type IV pili cluster 1	Holden *et al*. 2004
BPSL1814	*tad*-like type IV pili cluster 1	Holden *et al*. 2004
BPSL1815	*tad*-like type IV pili cluster 1	Holden *et al*. 2004
BPSL1816	*tad*-like type IV pili cluster 1	Holden *et al*. 2004
BPSL1817	*tad*-like type IV pili cluster 1	Holden *et al*. 2004
BPSL1818	*tad*-like type IV pili cluster 1	Holden *et al*. 2004
BPSL1819	*tad*-like type IV pili cluster 1	Holden *et al*. 2004
BPSL1820	*tad*-like type IV pili cluster 1	Holden *et al*. 2004
BPSL1821	*tad*-like type IV pili cluster 1	Holden *et al*. 2004
BPSL1891	*tad*-like type IV pili cluster 2	Holden *et al*. 2004
BPSL1892	*tad*-like type IV pili cluster 2	Holden *et al*. 2004
BPSL1893	*tad*-like type IV pili cluster 2	Holden *et al*. 2004
BPSL1894	*tad*-like type IV pili cluster 2	Holden *et al*. 2004
BPSL1895	*tad*-like type IV pili cluster 2	Holden *et al*. 2004
BPSL1896	*tad*-like type IV pili cluster 2	Holden *et al*. 2004
BPSL1897	*tad*-like type IV pili cluster 2	Holden *et al*. 2004
BPSL1898	*tad*-like type IV pili cluster 2	Holden *et al*. 2004
BPSL1899	*tad*-like type IV pili cluster 2	Holden *et al*. 2004
BPSL1900	*hypothetical protein*	Holden *et al*. 2004
BPSL1901	putative hemagglutinin-related transmembrane protein. Similar to BPSS1974	Holden *et al*. 2004
BPSL1902	putative hemagglutinin-related transmembrane protein. Similar to BPSL1901	Holden *et al*. 2004
BPSL2026	Type I fimbriae 4	Holden *et al*. 2004
BPSL2027	Type I fimbriae 4	Holden *et al*. 2004
BPSL2028	Type I fimbriae 4	Holden *et al*. 2004
BPSL2029	Type I fimbriae 4	Holden *et al*. 2004
BPSL2030	Type I fimbriae 4	Holden *et al*. 2004
BPSL2031	Type I fimbriae 4	Holden *et al*. 2004
BPSL2063	Hep_Hag family hemagglutinin-like protein	Holden *et al*. 2004
BPSL2094	*ompR*	Holden *et al*. 2004
BPSL2214	Putative NRPS cluster 2. Linked to putative efflux transport genes	Holden *et al*. 2004
BPSL2215	Putative NRPS cluster 2. Linked to putative efflux transport genes	Holden *et al*. 2004
BPSL2216	Putative NRPS cluster 2. Linked to putative efflux transport genes	Holden *et al*. 2004
BPSL2217	Putative NRPS cluster 2. Linked to putative efflux transport genes	Holden *et al*. 2004
BPSL2218	Putative NRPS cluster 2. Linked to putative efflux transport genes	Holden *et al*. 2004
BPSL2219	Putative NRPS cluster 2. Linked to putative efflux transport genes	Holden *et al*. 2004
BPSL2220	Putative NRPS cluster 2. Linked to putative efflux transport genes	Holden *et al*. 2004
BPSL2221	Putative NRPS cluster 2. Linked to putative efflux transport genes	Holden *et al*. 2004
BPSL2222	Putative NRPS cluster 2. Linked to putative efflux transport genes	Holden *et al*. 2004
BPSL2223	Putative NRPS cluster 2. Linked to putative efflux transport genes	Holden *et al*. 2004
BPSL2224	Putative NRPS cluster 2. Linked to putative efflux transport genes	Holden *et al*. 2004
BPSL2225	Putative NRPS cluster 2. Linked to putative efflux transport genes	Holden *et al*. 2004
BPSL2226	Putative NRPS cluster 2. Linked to putative efflux transport genes	Holden *et al*. 2004
BPSL2227	Putative NRPS cluster 2. Linked to putative efflux transport genes	Holden *et al*. 2004
BPSL2228	Putative NRPS cluster 2. Linked to putative efflux transport genes	Holden *et al*. 2004
BPSL2229	Putative NRPS cluster 2. Linked to putative efflux transport genes	Holden *et al*. 2004
BPSL2230	Putative NRPS cluster 2. Linked to putative efflux transport genes	Holden *et al*. 2004
BPSL2231	Putative NRPS cluster 2. Linked to putative efflux transport genes	Holden *et al*. 2004
BPSL2232	Putative NRPS cluster 2. Linked to putative efflux transport genes	Holden *et al*. 2004
BPSL2233	Putative NRPS cluster 2. Linked to putative efflux transport genes	Holden *et al*. 2004
BPSL2234	RND multi-drug efflux pump	Holden *et al*. 2004
BPSL2235	RND multi-drug efflux pump	Holden *et al*. 2004
BPSL2236	RND multi-drug efflux pump	Holden *et al*. 2004
BPSL2403	PLC-1, non-hemolytic phospholipase C precursor (EC:3.1.4.3) - secreted factor	Holden *et al*. 2004
BPSL2436	RpoE - attenuated (K96243)	Holden *et al*. 2004
BPSL2468	NorM multidrug efflux protein	Holden *et al*. 2004
BPSL2468	homologue of *norM,* a multidrug efflux pump that confers polymyxin resistance in *Burkholderia vietnamensis*	Holden *et al*. 2004
BPSL2672	WbiI (305) putative epimerase/dehydratase	Holden *et al*. 2004
BPSL2673	WbiH (305) putative glycosyl transferase	Holden *et al*. 2004
BPSL2674	WbiG (305) putative epimerase/dehydratase	Holden *et al*. 2004
BPSL2675	WbiF (305) putative glycosyl transferase	Holden *et al*. 2004
BPSL2676	WbiE (305) putative glycosyl transferase	Holden *et al*. 2004
BPSL2677	WbiD (305) putative glycosyl transferase	Holden *et al*. 2004
BPSL2678	WbiC (305) putative glycosyl transferase	Holden *et al*. 2004
BPSL2679	WbiB (305) putative epimerase/dehydratase	Holden *et al*. 2004
BPSL2680	WbiA (305) putative O-antigen acetylase	Holden *et al*. 2004
BPSL2681	Wzt - putative lipopolysaccharide ABC transporter, ATP-binding protein	Holden *et al*. 2004
BPSL2682	Wzm - putative lipopolysaccharide ABC transporter, permease protein	Holden *et al*. 2004
BPSL2683	rmlD	Holden *et al*. 2004
BPSL2684	rmlC	Holden *et al*. 2004
BPSL2685	rmlA	Holden *et al*. 2004
BPSL2686	rmlB	Holden *et al*. 2004
BPSL2687	apaH	Holden *et al*. 2004
BPSL2688	plsC	Holden *et al*. 2004
BPSL2708	Ambler class B b -lactamase	Holden *et al*. 2004
BPSL2752	Putative type IV (partial cluster including pilin)	Holden *et al*. 2004
BPSL2753	Putative type IV (partial cluster including pilin)	Holden *et al*. 2004
BPSL2754	Putative type IV (partial cluster including pilin)	Holden *et al*. 2004
BPSL2755	Putative type IV (partial cluster including pilin)	Holden *et al*. 2004
BPSL2756	Putative type IV (partial cluster including pilin)	Holden *et al*. 2004
BPSL2769	potentially involved in capsular polysaccharide biosynthesis and export	Holden *et al*. 2004
BPSL2770	potentially involved in capsular polysaccharide biosynthesis and export	Holden *et al*. 2004
BPSL2771	potentially involved in capsular polysaccharide biosynthesis and export	Holden *et al*. 2004
BPSL2772	potentially involved in capsular polysaccharide biosynthesis and export	Holden *et al*. 2004
BPSL2773	potentially involved in capsular polysaccharide biosynthesis and export	Holden *et al*. 2004
BPSL2774	potentially involved in capsular polysaccharide biosynthesis and export	Holden *et al*. 2004
BPSL2775	potentially involved in capsular polysaccharide biosynthesis and export	Holden *et al*. 2004
BPSL2776	potentially involved in capsular polysaccharide biosynthesis and export	Holden *et al*. 2004
BPSL2777	potentially involved in capsular polysaccharide biosynthesis and export	Holden *et al*. 2004
BPSL2778	potentially involved in capsular polysaccharide biosynthesis and export	Holden *et al*. 2004
BPSL2779	potentially involved in capsular polysaccharide biosynthesis and export	Holden *et al*. 2004
BPSL2780	potentially involved in capsular polysaccharide biosynthesis and export	Holden *et al*. 2004
BPSL2781	potentially involved in capsular polysaccharide biosynthesis and export	Holden *et al*. 2004
BPSL2787	wcbT	Holden *et al*. 2004
BPSL2788	wcbS	Holden *et al*. 2004
BPSL2789	wcbR	Holden *et al*. 2004
BPSL2790	WcbQ	Holden *et al*. 2004
BPSL2791	wcbP	Holden *et al*. 2004
BPSL2792	wcbO	Holden *et al*. 2004
BPSL2793	wcbN	Holden *et al*. 2004
BPSL2794	WcbM	Holden *et al*. 2004
BPSL2795	gmhA	Holden *et al*. 2004
BPSL2796	WcbL	Holden *et al*. 2004
BPSL2797	WcbK	Holden *et al*. 2004
BPSL2798	WcbJ	Holden *et al*. 2004
BPSL2799	wcbI	Holden *et al*. 2004
BPSL2800	WcbH	Holden *et al*. 2004
BPSL2801	WcbG	Holden *et al*. 2004
BPSL2802	WcbF	Holden *et al*. 2004
BPSL2803	WcbE	Holden *et al*. 2004
BPSL2804	Wzt2 - capsular polysaccharide export ABC transporter, ATP-binding protein	Holden *et al*. 2004
BPSL2805	Wzm - capsular polysaccharide export ABC transporter, permease protein	Holden *et al*. 2004
BPSL2806	WcbD	Holden *et al*. 2004
BPSL2807	WcbC	Holden *et al*. 2004
BPSL2808	WcbB	Holden *et al*. 2004
BPSL2809	WcbA	Holden *et al*. 2004
BPSL2810	ManC	Holden *et al*. 2004
BPSL2840	flavohaemoglobin *hmpA*	Holden *et al*. 2004
BPSL2866	*oxyR*	Holden *et al*. 2004
BPSL3008	putative type IV pilus assembly protein	Holden *et al*. 2004
BPSL3009	type IV pilus assembly protein PilC	Holden *et al*. 2004
BPSL3010	type IV prepilin leader peptide type M1 (EC:3.4.23.43, EC:2.1.1.-)	Holden *et al*. 2004
BPSS0067	phospholipase C	Holden *et al*. 2004
BPSS0067	PLC-3, phospholipase C precursor (EC:3.1.4.3) - secreted factor	Holden *et al*. 2004
BPSS0088	outer-membrane protein XadA, surface anchor family protein. Hep_Hag family hemagglutinin-like protein	Holden *et al*. 2004
BPSS0091	Type I fimbriae 5	Holden *et al*. 2004
BPSS0092	Type I fimbriae 5	Holden *et al*. 2004
BPSS0093	Type I fimbriae 5	Holden *et al*. 2004
BPSS0094	Type I fimbriae 5	Holden *et al*. 2004
BPSS0120	Type I fimbriae 6	Holden *et al*. 2004
BPSS0121	Type I fimbriae 6	Holden *et al*. 2004
BPSS0130	Putative polyketide synthase (PKS)	Holden *et al*. 2004
BPSS0262	Aac aminoglycoside acetyltransferase	Holden *et al*. 2004
BPSS0292	BpeE (K96243)	Holden *et al*. 2004
BPSS0293	BpeF (K96243)	Holden *et al*. 2004
BPSS0294	multidrug efflux system lipoprotein	Holden *et al*. 2004
BPSS0299	Putative PKS/NRPS cluster 3, linked to putative transport regulatory and genes	Holden *et al*. 2004
BPSS0300	Putative PKS/NRPS cluster 3, linked to putative transport regulatory and genes	Holden *et al*. 2004
BPSS0301	Putative PKS/NRPS cluster 3, linked to putative transport regulatory and genes	Holden *et al*. 2004
BPSS0302	Putative PKS/NRPS cluster 3, linked to putative transport regulatory and genes	Holden *et al*. 2004
BPSS0303	Putative PKS/NRPS cluster 3, linked to putative transport regulatory and genes	Holden *et al*. 2004
BPSS0304	Putative PKS/NRPS cluster 3, linked to putative transport regulatory and genes	Holden *et al*. 2004
BPSS0305	Putative PKS/NRPS cluster 3, linked to putative transport regulatory and genes	Holden *et al*. 2004
BPSS0306	Putative PKS/NRPS cluster 3, linked to putative transport regulatory and genes	Holden *et al*. 2004
BPSS0307	Putative PKS/NRPS cluster 3, linked to putative transport regulatory and genes	Holden *et al*. 2004
BPSS0308	Putative PKS/NRPS cluster 3, linked to putative transport regulatory and genes	Holden *et al*. 2004
BPSS0309	Putative PKS/NRPS cluster 3, linked to putative transport regulatory and genes	Holden *et al*. 2004
BPSS0310	Putative PKS/NRPS cluster 3, linked to putative transport regulatory and genes	Holden *et al*. 2004
BPSS0311	Putative PKS/NRPS cluster 3, linked to putative transport regulatory and genes	Holden *et al*. 2004
BPSS0417	putative polysaccharide biosynthetic operons 2	Holden *et al*. 2004
BPSS0418	putative polysaccharide biosynthetic operons 2	Holden *et al*. 2004
BPSS0419	putative polysaccharide biosynthetic operons 2	Holden *et al*. 2004
BPSS0420	putative polysaccharide biosynthetic operons 2	Holden *et al*. 2004
BPSS0421	putative polysaccharide biosynthetic operons 2	Holden *et al*. 2004
BPSS0422	putative polysaccharide biosynthetic operons 2	Holden *et al*. 2004
BPSS0423	putative polysaccharide biosynthetic operons 2	Holden *et al*. 2004
BPSS0424	putative polysaccharide biosynthetic operons 2	Holden *et al*. 2004
BPSS0425	putative polysaccharide biosynthetic operons 2	Holden *et al*. 2004
BPSS0426	putative polysaccharide biosynthetic operons 2	Holden *et al*. 2004
BPSS0427	putative polysaccharide biosynthetic operons 2	Holden *et al*. 2004
BPSS0428	putative polysaccharide biosynthetic operons 2	Holden *et al*. 2004
BPSS0429	putative polysaccharide biosynthetic operons 2	Holden *et al*. 2004
BPSS0481	hmqA, CoA ligase (EC:6.2.1.32)	Holden *et al*. 2004
BPSS0482	hmqB, hypothetical protein	Holden *et al*. 2004
BPSS0483	hmqC, beta-ketoacyl-ACP synthase	Holden *et al*. 2004
BPSS0484	hmqD, 3-oxoacyl-[acyl-carrier-protein] synthase III (EC 2.3.1.41) (IMGterm)	Holden *et al*. 2004
BPSS0485	hmqE, metallo-beta lactamase-related protein	Holden *et al*. 2004
BPSS0486	hmqF, non-ribosomal peptide synthetase	Holden *et al*. 2004
BPSS0487	hmqG, hypothetical protein	Holden *et al*. 2004
BPSS0581	Pyochelin siderophore biosynthesis cluster	Holden *et al*. 2004
BPSS0582	Pyochelin siderophore biosynthesis cluster	Holden *et al*. 2004
BPSS0583	Pyochelin siderophore biosynthesis cluster	Holden *et al*. 2004
BPSS0584	Pyochelin siderophore biosynthesis cluster	Holden *et al*. 2004
BPSS0585	Pyochelin siderophore biosynthesis cluster	Holden *et al*. 2004
BPSS0586	Pyochelin siderophore biosynthesis cluster	Holden *et al*. 2004
BPSS0587	Pyochelin siderophore biosynthesis cluster	Holden *et al*. 2004
BPSS0588	Pyochelin siderophore biosynthesis cluster	Holden *et al*. 2004
BPSS0624	RND multidrug efflux pump	Holden *et al*. 2004
BPSS0625	RND multidrug efflux pump	Holden *et al*. 2004
BPSS0643	*pmrA*	Holden *et al*. 2004
BPSS0663	probable adhesin; possible gene remnant	Holden *et al*. 2004
BPSS0687	attenuated - sensor kinase protein (EC:2.7.13.3)	Holden *et al*. 2004
BPSS0796	Hep_Hag family hemagglutinin-like protein	Holden *et al*. 2004
BPSS0803	*Bacillus cereus* hemolysin III	Holden *et al*. 2004
BPSS0885	luxI, N-acylhomoserine lactone synthase (EC:2.3.1.184)	Holden *et al*. 2004
BPSS0887	luxR, N-acylhomoserine lactone dependent regulatory protein	Holden *et al*. 2004
BPSS0908	Hep_Hag family hemagglutinin-like protein	Holden *et al*. 2004
BPSS0946	PenA; Ambler class A b -lactamase	Holden *et al*. 2004
BPSS0974	metalloprotease A MrpA	Holden *et al*. 2004
BPSS0993	*katB* (catalase)	Holden *et al*. 2004
BPSS0994	may be involved in the production and regulation of secondary metabolite	Holden *et al*. 2004
BPSS0995	may be involved in the production and regulation of secondary metabolite	Holden *et al*. 2004
BPSS0996	may be involved in the production and regulation of secondary metabolite	Holden *et al*. 2004
BPSS0997	may be involved in the production and regulation of secondary metabolite	Holden *et al*. 2004
BPSS0998	may be involved in the production and regulation of secondary metabolite	Holden *et al*. 2004
BPSS0999	may be involved in the production and regulation of secondary metabolite	Holden *et al*. 2004
BPSS1000	may be involved in the production and regulation of secondary metabolite	Holden *et al*. 2004
BPSS1001	may be involved in the production and regulation of secondary metabolite	Holden *et al*. 2004
BPSS1002	may be involved in the production and regulation of secondary metabolite	Holden *et al*. 2004
BPSS1003	may be involved in the production and regulation of secondary metabolite	Holden *et al*. 2004
BPSS1004	may be involved in the production and regulation of secondary metabolite	Holden *et al*. 2004
BPSS1005	may be involved in the production and regulation of secondary metabolite	Holden *et al*. 2004
BPSS1006	Putative antibiotic PKS/NRPS 5. Cluster contains putative resistance protein (BPSS1011). CDSs linked to the cluster (BPSS0994–BPSS1005) may be involved in the production	Holden *et al*. 2004
BPSS1007	Putative antibiotic PKS/NRPS 5. Cluster contains putative resistance protein (BPSS1011). CDSs linked to the cluster (BPSS0994–BPSS1005) may be involved in the production	Holden *et al*. 2004
BPSS1008	Putative antibiotic PKS/NRPS 5. Cluster contains putative resistance protein (BPSS1011). CDSs linked to the cluster (BPSS0994–BPSS1005) may be involved in the production	Holden *et al*. 2004
BPSS1009	Putative antibiotic PKS/NRPS 5. Cluster contains putative resistance protein (BPSS1011). CDSs linked to the cluster (BPSS0994–BPSS1005) may be involved in the production	Holden *et al*. 2004
BPSS1010	Putative antibiotic PKS/NRPS 5. Cluster contains putative resistance protein (BPSS1011). CDSs linked to the cluster (BPSS0994–BPSS1005) may be involved in the production	Holden *et al*. 2004
BPSS1011	Putative antibiotic PKS/NRPS 5. Cluster contains putative resistance protein (BPSS1011). CDSs linked to the cluster (BPSS0994–BPSS1005) may be involved in the production	Holden *et al*. 2004
BPSS1039	IrlS	Holden *et al*. 2004
BPSS1040	IrlR	Holden *et al*. 2004
BPSS1041	czcA; cation efflux system protein	Holden *et al*. 2004
BPSS1166	Putative antibiotic PKS/NRPS 6. Cluster contains putative efflux protein (BPSS1166). May be linked to the cluster below	Holden *et al*. 2004
BPSS1167	Putative antibiotic PKS/NRPS 6. Cluster contains putative efflux protein (BPSS1166). May be linked to the cluster below	Holden *et al*. 2004
BPSS1168	Putative antibiotic PKS/NRPS 6. Cluster contains putative efflux protein (BPSS1166). May be linked to the cluster below	Holden *et al*. 2004
BPSS1169	Putative antibiotic PKS/NRPS 6. Cluster contains putative efflux protein (BPSS1166). May be linked to the cluster below	Holden *et al*. 2004
BPSS1170	Putative antibiotic PKS/NRPS 6. Cluster contains putative efflux protein (BPSS1166). May be linked to the cluster below	Holden *et al*. 2004
BPSS1171	Putative antibiotic PKS/NRPS 6. Cluster contains putative efflux protein (BPSS1166). May be linked to the cluster below	Holden *et al*. 2004
BPSS1172	Putative antibiotic PKS/NRPS 6. Cluster contains putative efflux protein (BPSS1166). May be linked to the cluster below	Holden *et al*. 2004
BPSS1173	Putative antibiotic PKS/NRPS 6. Cluster contains putative efflux protein (BPSS1166). May be linked to the cluster below	Holden *et al*. 2004
BPSS1174	Putative antibiotic PKS/NRPS 6. Cluster contains putative efflux protein (BPSS1166). May be linked to the cluster below	Holden *et al*. 2004
BPSS1176	luxR,N-acyl-homoserine lactone dependent regulatory protein	Holden *et al*. 2004
BPSS1180	luxI, N-acylhomoserine lactone synthase	Holden *et al*. 2004
BPSS1181	Putative antibiotic PKS/NRPS 7. May be linked to the cluster above and a N-acyl homoserine lactone dependant quorum sensing regulon (BPSS1176–BPSS1180)	Holden *et al*. 2004
BPSS1182	Putative antibiotic PKS/NRPS 7. May be linked to the cluster above and a N-acyl homoserine lactone dependant quorum sensing regulon (BPSS1176–BPSS1180)	Holden *et al*. 2004
BPSS1183	Putative antibiotic PKS/NRPS 7. May be linked to the cluster above and a N-acyl homoserine lactone dependant quorum sensing regulon (BPSS1176–BPSS1180)	Holden *et al*. 2004
BPSS1184	Putative antibiotic PKS/NRPS 7. May be linked to the cluster above and a N-acyl homoserine lactone dependant quorum sensing regulon (BPSS1176–BPSS1180)	Holden *et al*. 2004
BPSS1185	Putative antibiotic PKS/NRPS 7. May be linked to the cluster above and a N-acyl homoserine lactone dependant quorum sensing regulon (BPSS1176–BPSS1180)	Holden *et al*. 2004
BPSS1187	Putative antibiotic PKS/NRPS 7. May be linked to the cluster above and a N-acyl homoserine lactone dependant quorum sensing regulon (BPSS1176–BPSS1180)	Holden *et al*. 2004
BPSS1190	Putative antibiotic PKS/NRPS 7. May be linked to the cluster above and a N-acyl homoserine lactone dependant quorum sensing regulon (BPSS1176–BPSS1180)	Holden *et al*. 2004
BPSS1191	Putative antibiotic PKS/NRPS 7. May be linked to the cluster above and a N-acyl homoserine lactone dependant quorum sensing regulon (BPSS1176–BPSS1180)	Holden *et al*. 2004
BPSS1192	Putative antibiotic PKS/NRPS 7. May be linked to the cluster above and a N-acyl homoserine lactone dependant quorum sensing regulon (BPSS1176–BPSS1180)	Holden *et al*. 2004
BPSS1193	Putative antibiotic PKS/NRPS 7. May be linked to the cluster above and a N-acyl homoserine lactone dependant quorum sensing regulon (BPSS1176–BPSS1180)	Holden *et al*. 2004
BPSS1194	Putative antibiotic PKS/NRPS 7. May be linked to the cluster above and a N-acyl homoserine lactone dependant quorum sensing regulon (BPSS1176–BPSS1180)	Holden *et al*. 2004
BPSS1195	Putative antibiotic PKS/NRPS 7. May be linked to the cluster above and a N-acyl homoserine lactone dependant quorum sensing regulon (BPSS1176–BPSS1180)	Holden *et al*. 2004
BPSS1196	Putative antibiotic PKS/NRPS 7. May be linked to the cluster above and a N-acyl homoserine lactone dependant quorum sensing regulon (BPSS1176–BPSS1180)	Holden *et al*. 2004
BPSS1197	Putative antibiotic PKS/NRPS 7. May be linked to the cluster above and a N-acyl homoserine lactone dependant quorum sensing regulon (BPSS1176–BPSS1180)	Holden *et al*. 2004
BPSS1198	Putative antibiotic PKS/NRPS 7. May be linked to the cluster above and a N-acyl homoserine lactone dependant quorum sensing regulon (BPSS1176–BPSS1180)	Holden *et al*. 2004
BPSS1199	Putative antibiotic PKS/NRPS 7. May be linked to the cluster above and a N-acyl homoserine lactone dependant quorum sensing regulon (BPSS1176–BPSS1180)	Holden *et al*. 2004
BPSS1266	Putative PKS/NRPS 8. Cluster contains putative efflux protein (BPSS1268). Cluster is next to putative alternative sigma factor (BPSS1275)	Holden *et al*. 2004
BPSS1267	Putative PKS/NRPS 8. Cluster contains putative efflux protein (BPSS1268). Cluster is next to putative alternative sigma factor (BPSS1275)	Holden *et al*. 2004
BPSS1268	Putative PKS/NRPS 8. Cluster contains putative efflux protein (BPSS1268). Cluster is next to putative alternative sigma factor (BPSS1275)	Holden *et al*. 2004
BPSS1269	Putative PKS/NRPS 8. Cluster contains putative efflux protein (BPSS1268). Cluster is next to putative alternative sigma factor (BPSS1275)	Holden *et al*. 2004
BPSS1270	Putative PKS/NRPS 8. Cluster contains putative efflux protein (BPSS1268). Cluster is next to putative alternative sigma factor (BPSS1275)	Holden *et al*. 2004
BPSS1271	Putative PKS/NRPS 8. Cluster contains putative efflux protein (BPSS1268). Cluster is next to putative alternative sigma factor (BPSS1275)	Holden *et al*. 2004
BPSS1272	Putative PKS/NRPS 8. Cluster contains putative efflux protein (BPSS1268). Cluster is next to putative alternative sigma factor (BPSS1275)	Holden *et al*. 2004
BPSS1273	Putative PKS/NRPS 8. Cluster contains putative efflux protein (BPSS1268). Cluster is next to putative alternative sigma factor (BPSS1275)	Holden *et al*. 2004
BPSS1274	Putative PKS/NRPS 8. Cluster contains putative efflux protein (BPSS1268). Cluster is next to putative alternative sigma factor (BPSS1275)	Holden *et al*. 2004
BPSS1275	putative alternative sigma factor	Holden *et al*. 2004
BPSS1390	TTS1	Holden *et al*. 2004
BPSS1391	TTS1	Holden *et al*. 2004
BPSS1392	TTS1	Holden *et al*. 2004
BPSS1393	TTS1	Holden *et al*. 2004
BPSS1394	TTS1	Holden *et al*. 2004
BPSS1395	TTS1	Holden *et al*. 2004
BPSS1396	TTS1	Holden *et al*. 2004
BPSS1397	TTS1	Holden *et al*. 2004
BPSS1398	TTS1	Holden *et al*. 2004
BPSS1399	TTS1	Holden *et al*. 2004
BPSS1400	TTS1	Holden *et al*. 2004
BPSS1401	TTS1	Holden *et al*. 2004
BPSS1402	TTS1	Holden *et al*. 2004
BPSS1403	TTS1	Holden *et al*. 2004
BPSS1404	TTS1	Holden *et al*. 2004
BPSS1405	TTS1	Holden *et al*. 2004
BPSS1406	TTS1	Holden *et al*. 2004
BPSS1407	TTS1 (TTS1 assay)	Holden *et al*. 2004
BPSS1408	TTS1	Holden *et al*. 2004
BPSS1434	Hep_Hag family hemagglutinin-like protein	Holden *et al*. 2004
BPSS1439	hemagglutinin protein, surface anchor family protein	Holden *et al*. 2004
BPSS1492	bimA_Bp_BPSS1492	Holden *et al*. 2004
BPSS1493	hyphothetical protein	Holden *et al*. 2004
BPSS1494	virG; two-component response regulator protein	Holden *et al*. 2004
BPSS1495	virA; two-component regulator sensor kinase	Holden *et al*. 2004
BPSS1496	Uncharacterized protein ImpB	Holden *et al*. 2004
BPSS1497	Uncharacterized protein ImpC	Holden *et al*. 2004
BPSS1498	hcp1 (Mary Burtnick)	Holden *et al*. 2004
BPSS1499	Uncharacterized protein similar to VCA0109	Holden *et al*. 2004
BPSS1500	Protein ImpG/VasA	Holden *et al*. 2004
BPSS1501	Uncharacterized protein ImpH/VasB	Holden *et al*. 2004
BPSS1502	Clp-type ATPase chaperone protein	Holden *et al*. 2004
BPSS1503	VgrG protein	Holden *et al*. 2004
BPSS1508	lipoprotein, putative	Holden *et al*. 2004
BPSS1509	Uncharacterized protein ImpJ/VasE	Holden *et al*. 2004
BPSS1510	Chemotaxis motB protein	Holden *et al*. 2004
BPSS1511	IcmF-related protein	Holden *et al*. 2004
BPSS1523	BicP	Holden *et al*. 2004
BPSS1524	BopA protein	Holden *et al*. 2004
BPSS1525	type III secretion target BopE	Holden *et al*. 2004
BPSS1526	BapC	Holden *et al*. 2004
BPSS1527	BapB	Holden *et al*. 2004
BPSS1528	hypothetical protein (HNS-like regulatory protein)	Holden *et al*. 2004
BPSS1529	BipD	Holden *et al*. 2004
BPSS1530	bprA (HNS-like regulatory protein)	Holden *et al*. 2004
BPSS1531	BipC	Holden *et al*. 2004
BPSS1532	BipB protein	Holden *et al*. 2004
BPSS1533	BicA	Holden *et al*. 2004
BPSS1534	bsaZ	Holden *et al*. 2004
BPSS1535	type III secretion system protein BsaY	Holden *et al*. 2004
BPSS1536	bsaX	Holden *et al*. 2004
BPSS1537	bsaW	Holden *et al*. 2004
BPSS1538	bsaV	Holden *et al*. 2004
BPSS1539	BsaU protein	Holden *et al*. 2004
BPSS1540	BsaT protein	Holden *et al*. 2004
BPSS1541	bsaS	Holden *et al*. 2004
BPSS1542	bsaR	Holden *et al*. 2004
BPSS1543	type III secretion system protein BsaQ	Holden *et al*. 2004
BPSS1544	bsaP	Holden *et al*. 2004
BPSS1545	type III secretion system protein BsaO	Holden *et al*. 2004
BPSS1546	bsaN	Holden *et al*. 2004
BPSS1547	TTS3 or *bsa*	Holden *et al*. 2004
BPSS1548	TTS3 or *bsa*	Holden *et al*. 2004
BPSS1549	TTS3 or *bsa*	Holden *et al*. 2004
BPSS1550	TTS3 or *bsa*	Holden *et al*. 2004
BPSS1551	TTS3 or *bsa*	Holden *et al*. 2004
BPSS1552	TTS3 or *bsa*	Holden *et al*. 2004
BPSS1569	N-acylhomoserine lactone-dependent regulatory protein	Holden *et al*. 2004
BPSS1570	luxI, N-acylhomoserine lactone synthase (EC:2.3.1.184)	Holden *et al*. 2004
BPSS1593	type IVB pilus biosynthesis protein	Holden *et al*. 2004
BPSS1594	type IVB type pili cluster	Holden *et al*. 2004
BPSS1595	major pilin subunit	Holden *et al*. 2004
BPSS1596	type IVB pilus biosynthesis protein	Holden *et al*. 2004
BPSS1597	type IVB pilus biosynthesis protein	Holden *et al*. 2004
BPSS1598	type IVB type pili cluster	Holden *et al*. 2004
BPSS1599	type IVB pilus biosynthesis protein	Holden *et al*. 2004
BPSS1600	type IVB pilus biosynthesis protein	Holden *et al*. 2004
BPSS1601	type IVB pilus biosynthesis protein	Holden *et al*. 2004
BPSS1602	type IVB type pili cluster	Holden *et al*. 2004
BPSS1613	TTS2	Holden *et al*. 2004
BPSS1614	TTS2	Holden *et al*. 2004
BPSS1615	TTS2	Holden *et al*. 2004
BPSS1616	TTS2	Holden *et al*. 2004
BPSS1617	TTS2	Holden *et al*. 2004
BPSS1618	TTS2	Holden *et al*. 2004
BPSS1619	TTS2	Holden *et al*. 2004
BPSS1620	TTS2	Holden *et al*. 2004
BPSS1621	TTS2	Holden *et al*. 2004
BPSS1622	TTS2	Holden *et al*. 2004
BPSS1623	TTS2	Holden *et al*. 2004
BPSS1624	TTS2	Holden *et al*. 2004
BPSS1625	TTS2	Holden *et al*. 2004
BPSS1626	TTS2	Holden *et al*. 2004
BPSS1627	TTS2	Holden *et al*. 2004
BPSS1628	TTS2	Holden *et al*. 2004
BPSS1629	TTS2	Holden *et al*. 2004
BPSS1631	Putative lipopeptide antibiotic NRPS cluster 9.	Holden *et al*. 2004
BPSS1632	Putative lipopeptide antibiotic NRPS cluster 9.	Holden *et al*. 2004
BPSS1633	Putative lipopeptide antibiotic NRPS cluster 9.	Holden *et al*. 2004
BPSS1634	Putative lipopeptide antibiotic NRPS cluster 9.	Holden *et al*. 2004
BPSS1727	similar to filamentous hemagglutinin FhaB and FhaB2	Holden *et al*. 2004
BPSS1805	Miscellaneous cluster, possibly involved in the biosynthesis of a nonproteinogenic amino acid	Holden *et al*. 2004
BPSS1806	Miscellaneous cluster, possibly involved in the biosynthesis of a nonproteinogenic amino acid	Holden *et al*. 2004
BPSS1807	Miscellaneous cluster, possibly involved in the biosynthesis of a nonproteinogenic amino acid	Holden *et al*. 2004
BPSS1808	Miscellaneous cluster, possibly involved in the biosynthesis of a nonproteinogenic amino acid	Holden *et al*. 2004
BPSS1809	Miscellaneous cluster, possibly involved in the biosynthesis of a nonproteinogenic amino acid	Holden *et al*. 2004
BPSS1810	Miscellaneous cluster, possibly involved in the biosynthesis of a nonproteinogenic amino acid	Holden *et al*. 2004
BPSS1811	Miscellaneous cluster, possibly involved in the biosynthesis of a nonproteinogenic amino acid	Holden *et al*. 2004
BPSS1812	Miscellaneous cluster, possibly involved in the biosynthesis of a nonproteinogenic amino acid	Holden *et al*. 2004
BPSS1813	Miscellaneous cluster, possibly involved in the biosynthesis of a nonproteinogenic amino acid	Holden *et al*. 2004
BPSS1814	Miscellaneous cluster, possibly involved in the biosynthesis of a nonproteinogenic amino acid	Holden *et al*. 2004
BPSS1815	Miscellaneous cluster, possibly involved in the biosynthesis of a nonproteinogenic amino acid	Holden *et al*. 2004
BPSS1825	putative polysaccharide biosynthetic operons 1	Holden *et al*. 2004
BPSS1826	putative polysaccharide biosynthetic operons 1	Holden *et al*. 2004
BPSS1827	putative polysaccharide biosynthetic operons 1	Holden *et al*. 2004
BPSS1828	putative polysaccharide biosynthetic operons 1	Holden *et al*. 2004
BPSS1829	putative polysaccharide biosynthetic operons 1	Holden *et al*. 2004
BPSS1830	putative polysaccharide biosynthetic operons 1	Holden *et al*. 2004
BPSS1831	putative polysaccharide biosynthetic operons 1	Holden *et al*. 2004
BPSS1832	putative polysaccharide biosynthetic operons 1	Holden *et al*. 2004
BPSS1833	putative polysaccharide biosynthetic operons 1	Holden *et al*. 2004
BPSS1834	putative polysaccharide biosynthetic operons 1	Holden *et al*. 2004
