# Supplementary material for: Genomic Characterization of Burkholderia pseudomallei Isolates Selected for Medical Countermeasures Testing: Comparative Genomics Associated with Differential Virulence
Source: PLoS One. 2015 Mar 24;10(3):e0121052. doi: 10.1371/journal.pone.0121052 (PMC4372212; doi:10.1371/journal.pone.0121052)
Supplement: S4 Table — (PDF) [file pone.0121052.s008.pdf]

S4 Table. Survival information over the course of BALB/c challenge studies for all strains challenged

	time (days)																				
strain	1	2	3	4	5	6	7	8	9	10	11	12	13	14	15	16	17	18	19	20	21
K96243	7	7	7	7	7	7	7	7	7	7	5	4	4	4	4	4	4	4	4	4	4
406e	7	7	7	7	7	6	6	6	6	4	3	3	1	1	1	1	0	0	0	0	0
1026b	7	7	7	7	7	7	7	7	7	6	5	5	4	4	4	4	4	4	4	4	4
1106a	7	7	7	7	7	7	7	7	7	7	7	7	7	7	7	7	7	7	7	7	7
MSHR305	7	7	7	7	7	7	7	7	7	5	4	3	2	2	2	2	2	2	2	2	2
MSHR668	7	7	7	5	1	0	0	0	0	0	0	0	0	0	0	0	0	0	0	0	0
MSHR5855	7	7	7	6	5	3	2	1	1	0	0	0	0	0	0	0	0	0	0	0	0
MSHR5858	7	7	7	7	7	7	7	6	6	6	6	6	6	6	6	6	6	6	6	6	6
10134a	7	7	7	6	5	3	3	3	2	1	0	0	0	0	0	0	0	0	0	0	0
10303a	7	7	7	7	7	6	6	6	6	6	6	6	6	5							
